# Factors Beyond Karstification Have Shaped the Population Structure of a Surface‐Dwelling Minnow (*Phoxinus lumaireul*) Able to Disperse Underground

**DOI:** 10.1111/eva.70104

**Published:** 2025-05-12

**Authors:** Susanne Reier, Peter Trontelj, Luise Kruckenhauser, Martin Kapun, Alexandra Wanka, Anja Palandačić

**Affiliations:** ^1^ Department of Evolutionary Biology University of Vienna Vienna Austria; ^2^ First Zoological Department Natural History Museum Vienna Vienna Austria; ^3^ Department of Biology, Biotechnical Faculty University of Ljubljana Ljubljana Slovenia; ^4^ Central Research Laboratories Natural History Museum Vienna Vienna Austria

**Keywords:** biodiversity conservation, ddRAD sequencing, Dinaric Karst, freshwater species, genetic water tracing, karst hydrology

## Abstract

The Dinaric Karst, a biodiversity hotspot, features complex surface and subterranean hydrological networks that influence aquatic species distribution. This study investigates how karst hydrology shapes the genetic structure of the surface‐dwelling minnow *Phoxinus lumaireul*, examining both large‐scale and small‐scale population patterns. Using mitochondrial DNA and genome‐wide single nucleotide polymorphism (SNP) data of 827 specimens of *P. lumaireul*, three hypotheses were tested: (1) karst underground water connections facilitate genetic connectivity within and across river systems, whereas non‐karst rivers exhibit genetic connectivity mostly within the same system; (2) historical and occasional hydrological connections have shaped present‐day population structure, leaving genetic signatures of relatedness where no contemporary hydrological links exist; and (3) genomic approaches provide additional insights into biologically relevant connections that may not be captured by classical tracing tests. The large‐scale analyses confirmed three main genetic groups (1a–c), whose structure was likely shaped by Pleistocene glaciations and associated microrefugia rather than by karst hydrology. Small‐scale structure analyses revealed that while karst hydrology facilitated gene flow within specific areas, connectivity was uneven and influenced by local hydrological dynamics and historical admixture events. Furthermore, some underground pathways identified by classical tracing tests lacked evidence of genetic connectivity, underscoring the limitations of traditional methods and the added value of genomic data in indirectly detecting biologically relevant hydrological connections. These findings highlight the influence of both historical processes and contemporary karst hydrology on *P. lumaireul* populations, emphasizing their vulnerability in karst ecosystems and the need for targeted conservation efforts.

## 1 Introduction

Understanding how different habitats influence biodiversity is crucial in ecological research and conservation genetics. Complex habitats provide diverse niches, reducing their overlap and enhancing overall diversity (e.g., Smith et al. 2014; Stein et al. 2014; Tokeshi and Arakaki 2012). Examples of highly structured habitats include karst systems, where geological formations interact with hydrological processes to create a mosaic of unique ecosystems (Ford and Williams 2007). One such system is the Dinaric Karst, a well‐developed karst region in the Western Balkan Peninsula characterized by limestone and dolomite formations (Zupan Hajna 2019). From 5 million to 2 million years ago (Mihevc 2001; Trontelj et al. 2007), the Dinaric Karst underwent intense karstification, a process in which water dissolves soluble rock (Bosak 2008), resulting in a complex landscape of sinking streams, sinkholes, and caves (Ford and Williams 2007) with hydrological connections between surface and subterranean water bodies (Bonacci 1987; Bonacci and Živaljević 1993; Legrand and Stringfield 1973). Identifying these underground connections, which facilitate water movement, nutrient transfer, but also pollutant dispersal, is a central challenge in karst hydrology research. The use of tracing tests using dyes or salts is the classical method and regarded as most effective for this purpose (e.g., Field 1999; Petrič et al. 2020). However, a promising approach involves assessing genetic subdivisions of populations on either side of hypothesized hydrological connections, providing insights into their historical development and suitability for aquatic taxa dispersal (Konec et al. 2016; Palandačić, Bonacci, et al. 2012; Reier, Kruckenhauser, et al. 2022).

Dinaric Karst is well known for its high levels of biodiversity and endemism of freshwater and subterranean fauna (e.g., Francuski et al. 2019; Freyhof and Brooks 2011; Gibert and Culver 2009; Marćić et al. 2021; Recknagel et al. 2023; Žganec et al. 2016), the consequence of numerous species evolving under unique conditions in restricted habitats, e.g., subterranean aquatic caves (e.g., Amin et al. 2019; Delić et al. 2020; Lukić et al. 2023; Sket 2012; Trontelj et al. 2007) or in surface water bodies located in karstic depressions (*polje*) (e.g., Bogutskaya and Zupancic 2003; Freyhof et al. 2006; Sket 1999). Vicariance and dispersal are emerging as key factors shaping genetic structure in karst regions (Delić et al. 2020; Trontelj 2019), with their relative importance varying from region to region (Culver et al. 2009). While karstification can create landscape barriers fostering small‐scale vicariance (Klobučar et al. 2013; Previšić et al. 2014), underground water connections may facilitate gene flow and connectivity among populations (Palandačić, Matschiner, et al. 2012; Zakšek et al. 2009). Fluctuations in groundwater levels further contribute to dispersal in karst areas (Katz et al. 2018; Reier, Bogutskaya, et al. 2022; Zakšek et al. 2009). Meanwhile, some genetic patterns may be remnants of older geological events predating karstification, such as tectonic activity, river captures, changes in flow direction, or the existence of prekarstic lake systems (Sket 2002; Verovnik et al. 2004; Žganec et al. 2016). The limited distributions of freshwater species in Dinaric Karst pose a threat to their survival, exacerbated by anthropogenic impacts such as water regulation (Fišer et al. 2022), pollution (Kačaroğlu 1999), and species introduction (Žganec et al. 2020). Therefore, understanding population connectivity through underground connections and water movement in karst aquifers is essential for biodiversity conservation efforts in the Dinaric Karst region.

The combination of older geological rearrangements and subsequent alternation of vicariance and dispersal, or both, during the formation of Dinaric Karst, seems also to have shaped the present genetic (sub)structures in the genus *Phoxinus* inhabiting the area (Palandačić et al. 2015; Reier, Bogutskaya, et al. 2022; Reier, Kruckenhauser, et al. 2022; Vučić et al. 2018). These small minnows belong to the family Leuciscidae and inhabit a variety of oxygen‐rich waters throughout Europe and Asia (Frost 1943; Tack 1940). Several species of *Phoxinus* inhabit the Dinaric Karst, with some being limited to a specific river drainage (e.g., endemic *P. krkae*; Bogutskaya et al. 2020) and others widespread, inhabiting Adriatic and Black Sea basins in the Karst and beyond, such as *P. lumaireul* (Palandačić et al. 2015; Reier, Bogutskaya, et al. 2022; Reier, Kruckenhauser, et al. 2022; Vučić et al. 2018). *Phoxinus lumaireul* exhibits a eurytopic characteristic that enables the species to thrive under varying conditions, including both karstic and non‐karstic streams (Reier, Kruckenhauser, et al. 2022).

Although anthropogenic impacts on the distribution of *Phoxinus* species have been reported previously (e.g., Denys et al. 2020; Miró and Ventura 2015; Museth et al. 2007), studies focusing on *P. lumaireul* have indicated a minor role of human translocations in shaping its current genetic structure (Palandačić et al. 2015, 2020). Instead, genetic patterns in this species are strongly influenced by natural hydrological dynamics, particularly in karst environments. The study by Reier, Kruckenhauser, et al. (2022) provides evidence for ongoing underground dispersal and historical water connections shaping the genetic structure of *P. lumaireul* in the North‐western (NW) Dinaric Karst. This was reflected in the higher genetic similarity observed among populations within the karst area compared to those in adjacent non‐karst regions (hereafter, “non‐karst” refers to areas located outside the geological boundaries of the Dinaric Karst, characterized by surface‐connected river systems [RSs] only). The study identified three mitochondrial (mtDNA) lineages (designated as groups 1a–c) with distribution ranges beyond the NW Dinaric Karst. These groups were also distributed irrespective of the Adriatic–Black Sea basin divide, with Adriatic haplotypes (group 1a) found in the Black Sea basin, suggesting historical or ongoing gene flow across drainage divides. Nevertheless, limited genetic markers and underrepresentation of non‐karst sampling sites have hindered a comprehensive understanding of the demographic processes underlying these patterns.

To address the questions in detail, this study incorporates both additional sampling from non‐karst areas and high‐resolution genome‐wide data. First, it evaluates whether the previously observed (Reier, Kruckenhauser, et al. 2022) three main genetic groups (1a–c) in *P. lumaireul* are supported by genomic data. Second, the influence of karst hydrology (contemporary and past) on the small‐scale population structure was tested by evaluating three hypotheses:

Hypothesis 1 *Karst underground water connections facilitate genetic connectivity within and between river systems, while non‐karst water systems restrict connectivity to surface‐linked populations*.

If karst RSs enable underground dispersal, genetic similarity should be high among sampling sites that are connected underground, even across RSs. Conversely, in non‐karst RSs, where connectivity is restricted to surface water networks, genetic similarity should be high only within individual RSs and low between them.

Hypothesis 2 *Historical and occasional hydrological connections have shaped present‐day population structure, leaving detectable genetic signatures even where no contemporary hydrological connection exists*.

If past hydrological events shaped population structure, then genetic analyses should detect signals of historical connectivity—such as introgression or shared ancestry—even in the absence of current hydrological links.

Hypothesis 3 *Genomic approaches complement traditional hydrological tracing by assessing the biological relevance of known hydrological connections*.

If *P. lumaireul* can disperse through underground pathways, then indications for gene flow should be observed between populations connected by known hydrological links.

## 2 Materials and Methods

### 2.1 Study Area, Study Organism, and Sampling

**FIGURE 1:**
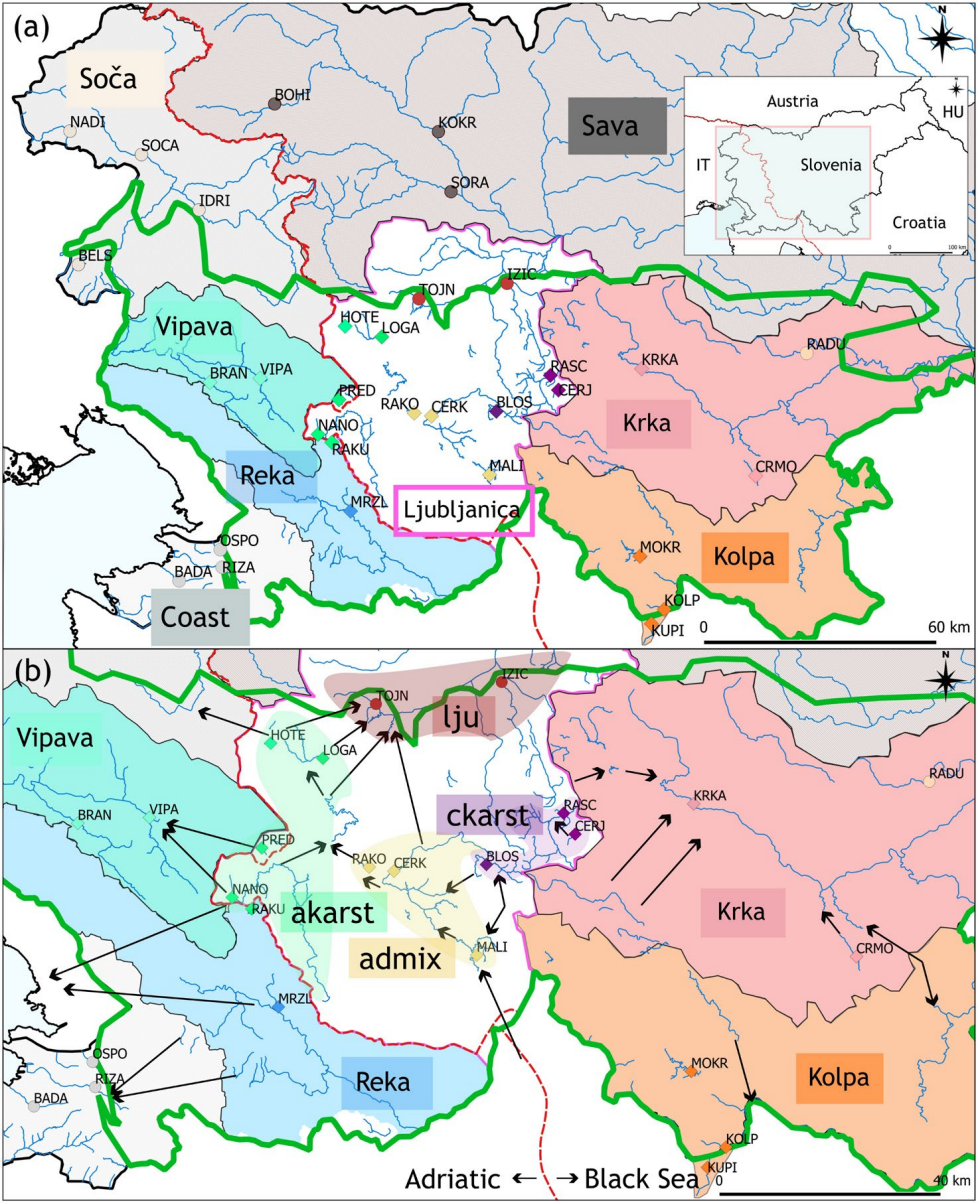
Study area in the north‐western Dinaric Karst (outlined in green) and adjacent regions, showcasing the distribution of sampling sites, represented as colored dots (non‐karst sampling sites) or diamonds (karst sampling sites). (a) View of 8 main river systems (RSs). Each RS is represented as shaded area with different colors. The Ljubljanica RS is outlined in pink. (b) Closer detail highlighting the karstic river systems and clusters identified within the Ljubljanica RS (akarst [green], admix [yellow], ckarst [purple], lju [dark red]) and the Krka RS (sampling site RADU, encircled in beige). Arrows depict underground connections between surface rivers pointing in the direction of flow. Red dashed line indicates the border of the Adriatic–Black Sea basins.

The study area (Figure 1a, outlined in green) is situated in the NW part of the Dinaric Karst. In 2020, the underground connections of this area were digitized by Petrič et al. (2020) with information from more than 200 tracing tests dating back more than 100 years. Thus, the hydrology of the area is well understood, with the western part draining toward the Adriatic Sea, and the center and south flowing to the Black Sea. A simplified scheme of connections is depicted in Figure 1b, including the divide between the two drainages (Figure 1a,b; red dashed line). There are five major RSs in the study area of the NW Dinaric Karst (Figure 1a, outlined in green): Vipava RSs (Figure 1a, turquoise colored) and Reka RSs (light blue) in the Adriatic Sea basin, and Ljubljanica RSs (outlined in pink), Krka RSs (light pink), and Kolpa RSs (orange) in the Black Sea basin (Gams 1993). Among the RSs, Ljubljanica is the most complex, comprising several sinking and underground karst rivers, as well as many surface and underground karst features (Gabrovšek and Turk 2011). In this area, *P. lumaireul* from 22 sampling sites within the territory of Slovenia and one in Croatia (Table 1) were collected.

**TABLE 1:** *Phoxinus lumaireul* sampling sites, by location on the Adriatic or Black Sea drainage basin. The site and the corresponding river system are included as an abbreviation, including the number of samples per population used for analysis and genetic clusters from the Ljubljanica river system (Figure 1b). Whether the site is from a karst area or not, and the type of hydrological connectivity (surface connection or exclusively underground) is indicated, along with the genetic group (1a–c).

River system	ID sampling site	Number of samples	Dinaric Karst	Connection type	Group
***Adriatic Sea***					
Coast	BADA	15	No	Surface and underground	1a
	OSPO	29			
	RIZA	37			
Soča	BELS	11	No	Surface	1a
	IDRI	29			
	NADI	30			
	SOCA	47			
Reka	MRZL	29	Yes	Underground	1a
Vipava	BRAN	31	Yes	Surface and underground	1a
	VIPA	38			
***Black Sea***					
Ljubljanica—admix	CERK	40	Yes	Underground	1a, 1c
	MALI	37			
	RAKO	19			
Ljubljanica—akarst	HOTE	19	Yes	Underground	1a
	LOGA	20			
	PRED	33			
	NANO	42			
	RAKU	26			
Ljubljanica—ckarst	BLOS	29	Yes	Underground	1c
	CERJ	36			
	RASC	17			
Ljubljanica—lju	TOJN	20	No	Surface and underground	1c
	IZIC	25			
Krka	CRMO	19	Yes	Surface and underground	1c
	KRKA	32			
	RADU	43			
Sava	BOHI	35	No	Surface	1c
	KOKR	19			
	SORA	33			
Kolpa	KOLP	34	Yes	Surface	1b
	KUPI	25			
	MOKR	35			

To assess the difference in genetic connectivity of *P. lumaireul* between karst and non‐karst areas, 10 sampling sites (Table 1) of the neighboring (non‐Dinaric Karst) area were also collected and included in the study (Figure 1a, all grayish colored). These samples were collected from the Soča RS in the Adriatic Sea basin and the coastal streams flowing directly into the Adriatic Sea (designated herein as Coast). In addition, populations from tributaries of the Sava RS in the Black Sea basin were also included (Figure 1).

In total, the DNA of 895 specimens of the species *P. lumaireul* from 32 sampling sites was analyzed (Table S1). Of these, 523 specimens were collected in previous studies, mostly from karst RSs (Palandačić et al. 2015; Reier, Kruckenhauser, et al. 2022) (see Table S1 for details). Additionally, fin clips of 372 specimens of *P. lumaireul* from eight, mostly non‐karst, sampling sites were collected in August 2021 for this study and stored in 96% EtOH prior to further analysis. All freshly collected fish specimens were acquired in accordance with national regulations and appropriate permits.

### 2.2 DNA Extraction and Sanger Sequencing

Genomic DNA of the 372 freshly collected samples was extracted from fin clips using the QIAmp DNeasy Blood and Tissue Kit (QIAGEN, Hilden, Germany) following the manufacturer's protocol.

The barcoding region of the mitochondrial cytochrome c oxidase subunit I (COI) gene was amplified from 255 of the freshly collected specimens using the primer pairs FishF1/FishR1 (Behrens‐Chapuis et al. 2015) and the polymerase chain reaction (PCR) protocol described in Palandačić et al. (2017). Sanger sequencing was conducted in one direction at Microsynth (Balgach, Switzerland) using the FishF1 forward primer.

### 2.3 Double‐Digest RAD Sequencing Data Sets

#### 2.3.1 Library Preparation and Sequencing

Double‐digest restriction site‐associated (ddRAD) sequencing libraries were prepared following the protocols outlined in Peterson et al. (2012) and Paulus et al. (2022), with a few custom modifications. The amount of input DNA ranged from 100 to 200 ng, and based on the DNA concentration, samples were organized into batches of 16. The protocol began with restriction digestion, conducted at 37°C for 2 h, using a mastermix comprising 3 μL of rCutSmart buffer from New England BioLabs (NEB), 0.3 μL *MSpI* (NEB), 0.3 μL *EcoRI* (NEB), 1.4 μL nuclease‐free water (NFW), and 25 μL DNA. Subsequently, the samples were cleaned with 1.2× magnetic beads (AmpliClean Cleanup kit, Nimagen), followed by rinsing twice with 80% EtOH and a final elution in 21 μL NFW. To attach adaptors for the specific restriction enzymes, we ligated 31.6 μM *MSpI* and 2 μM *EcoRI* adaptors to 19 μL digested DNA. This ligation step comprised 3 μL of each adaptor, 3 μL 10× T4 ligase buffer, 0.75 μL T4 Ligase (NEB), and 1.25 μL NFW. Adaptors were tagged with unique barcodes to ensure a distinct barcode combination for each sample within every batch of 16 samples. The ligation was conducted at 22°C for 1 h and heat‐terminated at 65°C for 10 min. Subsequently, the 16 samples from each batch were pooled and cleaned up using 1.2× magnetic beads, followed by two washes with 80% EtOH and final elution in 30 μL TE buffer. A crucial size selection step was implemented to narrow the fragment range to between 250 and 450 bp. This range had been previously determined in silico using ddRADseqTools (v0.42, Mora‐Márquez et al. 2017). Size selection was undertaken on a BluePippin instrument (Sage Science) utilizing a 2% agarose gel cassette and V1 marker. Subsequently, four PCR replicates were carried out for each pool to minimize potential PCR amplification bias. PCR reactions were performed with 5 μL of NEBNext Q5 Hot Start HiFi PCR Master Mix (NEB), 0.5 μL of each primer, and 4 μL of the library with 10 cycles. The forward and reverse primers included eight distinct 8‐bp long indices, which were combined to assign a unique combination to each pool. The four replicates of each pool were then combined and cleaned using 1× volume of magnetic beads, with elution in 16 μL NFW. DNA concentration was determined using a Qubit fluorometer (Thermo Fisher Scientific), while the mean fragment size was assessed using a TapeStation (Agilent). Finally, within each library, 16 pools were combined at equal molarity, resulting in a total of five libraries, which were sent to Macrogen Europe for sequencing on five Illumina HiSeqX lanes, generating 100‐bp paired‐end reads.

#### 2.3.2 ddRAD Data Processing

Raw reads were first pre‐demultiplexed by Macrogen and subsequently trimmed with trim‐galore v0.6 (Krueger et al. 2023) using a quality threshold of 30 and minimum length filter of 40 bp. STACKS v2.59 (Rochette et al. 2019) was used to remove potential PCR duplicates (function *clone_filter*) and to demultiplex the individuals by barcodes (*process_radtags*). Each demultiplexed individual was aligned to a reference genome of *P. phoxinus* (PRJNA1030283; Oriowo et al. 2025) using bwa‐mem v0.7.13 (Li 2013) and default parameters. The output bam files were further sorted by reference position with SAMtools v1.12 (Danecek et al. 2021). A catalog file of loci was built with the *gstacks* program implemented in STACKS v2.59. Following filtering criteria for single nucleotide polymorphisms (SNP) were applied using the program *populations* implemented in STACKS v2.59: SNPs were filtered with −r 0.5 (SNP present in at least 50% of individuals per population), −R 0.85 (SNPs present in at least 85% of populations), and ‐‐min‐maf 0.03 (minor allele frequency of 3%). Individuals with a significant amount of missing data (> 50%, assessed with “‐‐missing‐indv”) were removed from the dataset using vcftools v0.1.13 (Danecek et al. 2011). The final dataset was further filtered using only the remaining individuals to eliminate SNPs with missing data exceeding a threshold of 15% per site (command “‐‐max‐missing 0.85”) and a minimum mean depth of 5 (“‐‐min‐meanDP”) in vcftools v0.1.13. The filtered datasets were further processed with plink2 (Chang et al. 2015) to prune for linkage disequilibrium: SNPs were pruned using a sliding window of 100 SNPs, a step size of 10, and an *r* ^2^ threshold of 0.7 (“‐‐indep‐pairwise 100 10 0.7”).

For historical analyses (related to Hypothesis 2), we re‐ran populations in STACKS without the samples exceeding 50% missing data to ensure high‐quality datasets. Using more SNPs enhances statistical power and provides small‐scale insights into population structure essential for tools like TreeMix, Dsuite, Stairway Plot, and fineRADstructure (Excoffier et al. 2021; Malinsky, Trucchi, et al. 2018; Malinsky et al. 2021; Pickrell and Pritchard 2012). As there were still individuals with a high percentage of missing data in the dataset (> 60%), these individuals were excluded for the historical analyses. The final datasets were further filtered to eliminate SNPs with missing data exceeding a threshold of 5% per site (command “‐‐max‐missing 0.95”) using vcftools v0.1.13 (Danecek et al. 2011). Details about included population structure for each analysis are provided below in Section 2.5.2.

### 2.4 Evaluating Large‐Scale Population Structure

To investigate the large‐scale genetic structure of *P. lumaireul* across the NW Dinaric Karst, both mtDNA and genome‐wide SNP data were analyzed. More precisely, the three main genetic groups (1a–c) previously identified by Reier, Kruckenhauser, et al. (2022) were reassessed based on an extended dataset (including additional non‐karst sampling sites. These analyses offer information on broader phylogeographic patterns.

#### 2.4.1 mtDNA Analysis

To assess historical maternal lineage structure, COI sequences were analyzed. Sequences underwent manual verification, trimming, and alignment without ambiguity (no missing data) in Geneious v2.10.3 (https://www.geneious.com). Newly generated sequences were combined with existing COI sequences from previous studies (Palandačić et al. 2015; Reier, Kruckenhauser, et al. 2022) (see Table S1 for details) to create a comprehensive dataset of 749 sequences. To visualize large‐scale population patterns and lineage distribution, a median‐joining (MJ) haplotype network (Bandelt et al. 1999) was computed using PopART v1.7 software (Leigh and Bryant 2015).

#### 2.4.2 Principal Component Analysis

A principal component analysis (PCA) was conducted to test whether the three large scale groups (1a–c) remain distinct in SNP‐based analyses and to assess genetic differences between karst and non‐karst populations using the R package SNPRelate (Zheng et al. 2012). All R analyses in this study were conducted in R v4.2.2 (R Core Team 2023).

#### 2.4.3 Effective Population Size History

To reconstruct historical demographic fluctuations and infer potential population expansions and contractions (bottlenecks), effective population size (*N*_e_) dynamics were modeled using Stairway plot v2.1 (Liu and Fu 2020) using the diversity dataset. This analysis was conducted separately for the three main genetic groups (1a–c; see Section 3). The Folded Site Frequency Spectrum (SFS) for each population was generated using the python script easySFS (https://github.com/isaacovercast/easySFS/; accessed on 29 October 2023). A preview of the data was initially generated to identify values for projecting down each group to account for missing data, but at the same time with the goal of maximizing the number of segregating sites (Gutenkunst et al. 2009). After running the script for projection, the SFS values obtained were imported into a blueprint file for Stairway Plot v2.1. The parameter settings for Stairway Plot were configured with 67% of SFSs used for training with four random breakpoints set at (*n*−2)/4, (*n*−2)/2, (*n*−2) × (3/4), and *n*−2 as suggested by (Liu and Fu 2020), whereby n corresponds to the number of SFSs. Additionally, a generation time of 2+ years was applied, considering that the age of the individuals typically does not exceed 3+ years (Altinişik and Yağlioğlu 2022; Frost 1943; Mills 1987; Nunn et al. 2007; Saç and Özuluğ 2020). No mutation rate of *Phoxinus* is available, and therefore, a rate of 3.51 × 10^−9^ per site per generation was used (Tian et al. 2022; Oriowo et al. 2025).

### 2.5 Evaluating Small‐Scale Population Structure

While large‐scale population structure analyses were conducted to reveal broad genetic patterns shaped primarily by historical processes, small‐scale analyses are needed to analyze the influence of contemporary hydrological features and past admixture events on a local scale. To assess how possible differences between karst and non‐karst hydrology may influence gene flow, detailed genetic analyses focusing on both present‐day and historical connectivity were conducted.

#### 2.5.1 Evaluating Genetic Connectivity in Karst and Non‐Karst River Systems

##### 2.5.1.1 ADMIXTURE Analysis

To compare genetic connectivity between karst and non‐karst RSs, a likelihood‐based clustering approach was applied using ADMIXTURE (Alexander et al. 2009). If karst underground connections facilitate genetic connectivity between *P. lumaireul* from different sampling sites, then populations within and across karst RSs should exhibit higher admixture proportions, indicating gene flow beyond surface hydrological boundaries. Conversely, if non‐karst RSs restrict dispersal to surface connections, then admixture should be limited to populations within the same RS, with distinct genetic clusters forming between different RSs, following Hypothesis 1.

The analysis was performed with the output bim‐file of plink2 (Chang et al. 2015), which was adjusted for compatibility with the software ADMIXTURE v1.3.0 (Alexander et al. 2009), by editing chromosome identifiers as needed. ADMIXTURE was run for K values ranging from 1 to 18, with 10 runs conducted for each K. The optimal K was evaluated using cross‐validation (CV) error values, a metric provided by ADMIXTURE to assess model performance. Membership coefficients for each K were visualized as bar charts using BITEV2 (Milanesi et al. 2017) in R. Additionally, the model fit for each K was evaluated using evalAdmix (Garcia‐Erill and Albrechtsen 2020). When individuals deviate from the model, those with comparable demographic backgrounds or relatedness, typically from the same population, will exhibit a positive correlation. Conversely, individuals with distinct histories are represented as having common ancestral populations as sources of admixture will display a negative correlation (Garcia‐Erill and Albrechtsen 2020).

##### 2.5.1.2 Contemporary Migration Rates

To assess the extent of recent gene flow within and between RSs, BA3‐SNPs v3.0.4 (Mussmann et al. 2019), a SNP‐based adaptation of BayesAss (Wilson and Rannala 2003), was employed to estimate contemporary migration rates (*m*). Separate analyses were performed for the Adriatic Sea and Black Sea basins. Three sampling sites from the Ljubljanica RS (CERK, RAKO, MALI), which showed admixed ancestries between Adriatic 1a and Black Sea 1c groups (see Section 2.5.1.1) were included in both analyses to detect cross‐drainage connectivity. If underground connections facilitate dispersal, significant migration rates should be detected both within individual karst RSs and between separate karst RSs, whereas non‐karst systems should only exhibit gene flow within the same RS, as expected in Hypothesis 1. If significant migration rates are detected between populations connected by underground pathways, as identified through classical tracing tests, this supports the biological relevance for fish dispersal of these hydrological connections, aligning with Hypothesis 3.

The analysis was performed with 10 repetitions in BA3‐SNP‐autotune (Mussmann et al. 2019) in order to determine optimal mixing parameters for migration rate (*δ*M), allele frequencies (*δ*A), and inbreeding coefficients (*δ*F). For the Adriatic Sea basin, the parameters were set to *δ*M = 0.10, *δ*A = 0.55, and *δ*F = 0.10. For the Black Sea basin, the parameters were *δ*M = 0.10, *δ*A = 0.325, and *δ*F = 0.0375. Subsequently, BA3‐SNPs analyses were executed with the specified mixing parameters for 50 million generations, sampling every 100th generation and the first 5 million generations discarded as burn‐in. The convergence of Markov chain Monte Carlo chains was evaluated using Tracer v1.7.1 (Rambaut et al. 2018). Migration rates (m) were determined to be statistically significant if the 95% credible intervals (mean migration rate ± 1.96 × mean standard deviation) did not include zero. Hereby, only migration rates exceeding 0.01 were considered. Additionally, *p*‐values were calculated for the migration rates to test for significant deviations from the null hypothesis of no migration. Accordingly, migration rates with *p* < 0.05 were considered significant. The R package circlize v0.4.15 (Gu et al. 2014) was utilized to visualize the direction and magnitude of gene flow.

##### 2.5.1.3 Pairwise *F*_ST_

To quantify genetic differentiation within and among RSs, pairwise *F*_ST_ values between sampling sites were calculated. If karst hydrology allows for increased connectivity, lower *F*_ST_ values should be observed within and across karst RSs compared to non‐karst systems, where higher *F*_ST_ values should indicate genetic differentiation between different RSs, following Hypothesis 1. Additionally, low *F*_ST_ values between sampling sites with known underground connections provide further support for Hypothesis 3, indicating that hydrological links are reflected in genetic structure.

The analysis was performed using the pairwise.WCfst() function of the R package hierfstat v0.5‐11 (Goudet and Jombart 2022), which implements Weir and Cockerham's estimator (Weir and Cockerham 1984). The resulting pairwise *F*_ST_ matrix provided a measure of genetic differentiation between each pair of sampling sites. The *F*_*ST*_ matrix was visualized as a heatmap using the R package ComplexHeatmap v2.18 (Gu 2022; Gu et al. 2016).

##### 2.5.1.4 Genetic Diversity and Connectivity

To assess whether genetic structure aligns with hydrological connectivity, a set of descriptive population genetics statistics, including mean observed (*H*_O_) and expected (*H*_E_) heterozygosity, nucleotide diversity (π), and inbreeding coefficient (*F*_IS_), and their corresponding standard errors (SE), were calculated with the program populations of the STACKS pipeline (v2.59) for each separate sampling site. The output from STACKS was imported into R for further processing and visualization. Diversity metrics (*H*o, *H*e, π, *F*_IS_) were extracted from the STACKS output. These values were analyzed across the following population groupings (for details see Section 3) to evaluate how genetic diversity patterns correspond to hydrological networks: (i) main genetic groups (1a‐c) to assess large‐scale differences in genetic diversity; (ii) RSs and clusters in Ljubljanica RS (akarst, admix, ckarst, lju) as well as RADU sampling site in Krka RS, to examine small‐scale structure and potential hydrological influences; (iii) karst versus non‐karst status, to determine whether genetic diversity differs between karst and non‐karst hydrological environments; and (iv) hydrological underground connectivity (populations with confirmed underground water connections versus those without known connections based on tracing tests, see Table 1), to test whether hydrological connectivity influences genetic diversity. Boxplots were created to visualize diversity metrics across all categories. To assess differences in nucleotide diversity (π) among the previously described categories, pairwise Wilcoxon Rank‐Sum tests (Wilcoxon 1945) were conducted using the R package stats (R Core Team 2023). This nonparametric test was selected as it does not assume a normal distribution of the data, which was confirmed to be appropriate after testing for normality using the Shapiro–Wilk test (Shapiro and Wilk 1965), as implemented in the stats package. To account for multiple comparisons, *p*‐values were adjusted using the Bonferroni correction in the same package.

##### 2.5.1.5 Partitioning Genetic Variation

To determine whether genetic variation is structured according to hydrological connectivity, analysis of molecular variance (AMOVA) was conducted to examine the genetic variation among (i) the three main groups (1a–c); (ii) RSs and clusters in Ljubljanica RS (akarst, admix, ckarst, lju) and sampling site RADU in Krka RS (see Section 3); and (iii) rivers with surface connections and rivers with underground connections (see Table 1, Figure 1b). The three distinct sampling site structures utilized for the different AMOVAs are reported in Table S2. (i) If genetic variation is primarily structured by historical large‐scale processes, then AMOVA should show that the largest proportion of genetic variation is explained among the three main genetic groups (1a–c). (ii) If contemporary RSs play a dominant role, then a significant proportion of variation should be explained among RSs and clusters within Ljubljanica RS (see Section 3), indicating that genetic structure aligns with present‐day river networks. (iii) If population structure is primarily shaped by karst hydrology, then AMOVA should reveal that the largest proportion of genetic variance is explained by differences between populations with confirmed underground hydrological connections and those without such connections, rather than by differences among individual RSs.

AMOVA calculations were carried out with the software Arlequin v3.5 (Excoffier and Lischer 2010). Significance of fixation indices was evaluated through 10,000 random permutation procedures.

#### 2.5.2 Assessing the Impact of Historical Hydrological Connections on Population Structure

If historical hydrological connections existed, which enabled gene flow between populations, genetic traces of past admixture should still be detectable, even in the absence of present‐day connectivity. Such historical exchange would be reflected in patterns of co‐ancestry, introgression, and historical gene flow events. To examine to which extent the genetic structure of *P. lumaireul* was influenced by these effects, small‐scale genomic analyses were conducted.

##### 2.5.2.1 Co‐Ancestry Analysis With fineRADstructure

To assess s‐scale genetic relationships and past connectivity, RADpainter and fineRADstructure analysis (Malinsky, Trucchi, et al. 2018) were conducted, a modification of fineSTRUCTURE and ChromoPainter (Lawson et al. 2012), tailored for RAD data and non‐model organisms, to infer co‐ancestry matrices. The analysis used default parameters recommended by the developers (Malinsky, Trucchi, et al. 2018), and the results were plotted using the provided Rscript FinestructureLibrary.R (https://github.com/millanek/fineRADstructure/blob/master/FinestructureLibrary.R, accessed 29 December 2023).

##### 2.5.2.2 Historical Introgression With Dsuite

To detect historical gene flow events and assess whether hydrological shifts influenced past connectivity, Dsuite v.0.4 (Malinsky et al. 2021) was used to calculate Patterson's *D*, the *f*_4_‐ratio (Patterson et al. 2012), and *f*‐branch (*f*_b_) statistics (Malinsky, Svardal, et al. 2018). The test was conducted for all possible population trios to assess whether historical introgression occurred between distinct genetic groups. Sampling site clustering for the Dsuite analysis was determined based on previous analyses (clusters determined in ADMIXTURE and fineRADstructure) and described further under Results. RSs and clusters showing signs of admixture in the ADMIXTURE analysis were excluded from the dataset, as they would bias the estimation by introducing signals of recent introgression (Secci‐Petretto et al. 2023). Two individuals of *P. phoxinus* (mitochondrial clade 10 *sensu* Palandačić et al. (2017)) were used as an outgroup (Table S1). The dataset was generated employing identical parameters to those used for the TreeMix analysis (see below) through the program populations (STACKS), with the inclusion of two outgroup individuals of *P. phoxinus* (Table S1). The analysis used block sizes of 399 variants to obtain 20 jackknife blocks. Assignment of signals of introgression to specific branches of a given population tree was undertaken with the use of the *f*_b_ metric based on the outcomes of the *f*_4_‐ratio analysis (Malinsky et al. 2021). The input population topology used as the guide tree for the *f*_b_ estimation was calculated by executing 1000 bootstrapping runs in TreeMix v1.13 (Pickrell and Pritchard 2012), with the resulting trees summarized in a maximum‐likelihood (ML) consensus tree with the program consens in PHYLIP v3.698 (https://evolution.genetics.washington.edu, accessed on 20 October 2023).

##### 2.5.2.3 Historical Relationships Using TreeMix

To infer historical gene flow events and reconstruct past connectivity patterns, TreeMix v1.13 (Pickrell and Pritchard 2012) was used to estimate a ML tree of population relationships among the clusters identified by the population structure analyses described above. The same grouping as for the Dsuite analysis was used (see also Section 3), although this analysis allows for admixed groups (identified by ADMIXTURE analysis, see Section 3.3.1.) to also be included. The analysis was conducted allowing for 0–10 migration events per 100 runs and block size of 300 SNPs (parameter *‐k* 300). Standard errors for migration rates were calculated using the “‐se” option, and, subsequent to the addition of all populations, a round of global rearrangements was executed using the “‐global” option. No root was specified for the tree. Furthermore, the R package OptM v0.1.6 (Fitak 2021) was used to evaluate the best number of migration events, while TreeMix v1.13 was run again with 1000 replications, sampling each tree to construct an ML consensus tree using Consense in PHYLIP v3.698 (https://evolution.genetics.washington.edu/, accessed on 20 October 2023). Once obtained, the ML consensus tree was used as input for an additional TreeMix run with the evaluated optimal number of migration events. The R package BITE v2 (Milanesi et al. 2017) was used to plot the final tree with migration edges, with heatmaps of the residuals showing deviations from the model fit to the data and genetic drift between populations.

## 3 Results

### 3.1 ddRAD Datasets Preparation

For the ddRAD dataset, 895 individuals from 32 sites were sequenced. However, 68 samples were of poor quality and/or showed excessive missing data and were therefore excluded from subsequent analyses (Table S1).

These excluded samples were distributed across sampling sites, with the exception of sampling site MRZL (Reka RS), which exhibited a disproportionate number of low‐quality samples. Despite these exclusions, the remaining specimens per sampling site were sufficient for meaningful analyses (Table S1). The final dataset included 827 individuals and 4047 SNPs.

For the historical datasets, an additional 105 individuals were excluded due to excessive missing data (> 15%) in the high‐quality SNP dataset, resulting in 722 individuals available for analysis. After filtering, the final datasets included 8085 SNPs for TreeMix and fineRADstructure. The final dataset for Dsuite included 594 individuals and 8003 SNPs. Missing data per site were limited to less than 5%.

### 3.2 Large‐Scale Population Structure Across the Study Area

#### 3.2.1 Mitochondrial DNA

**FIGURE 2:**
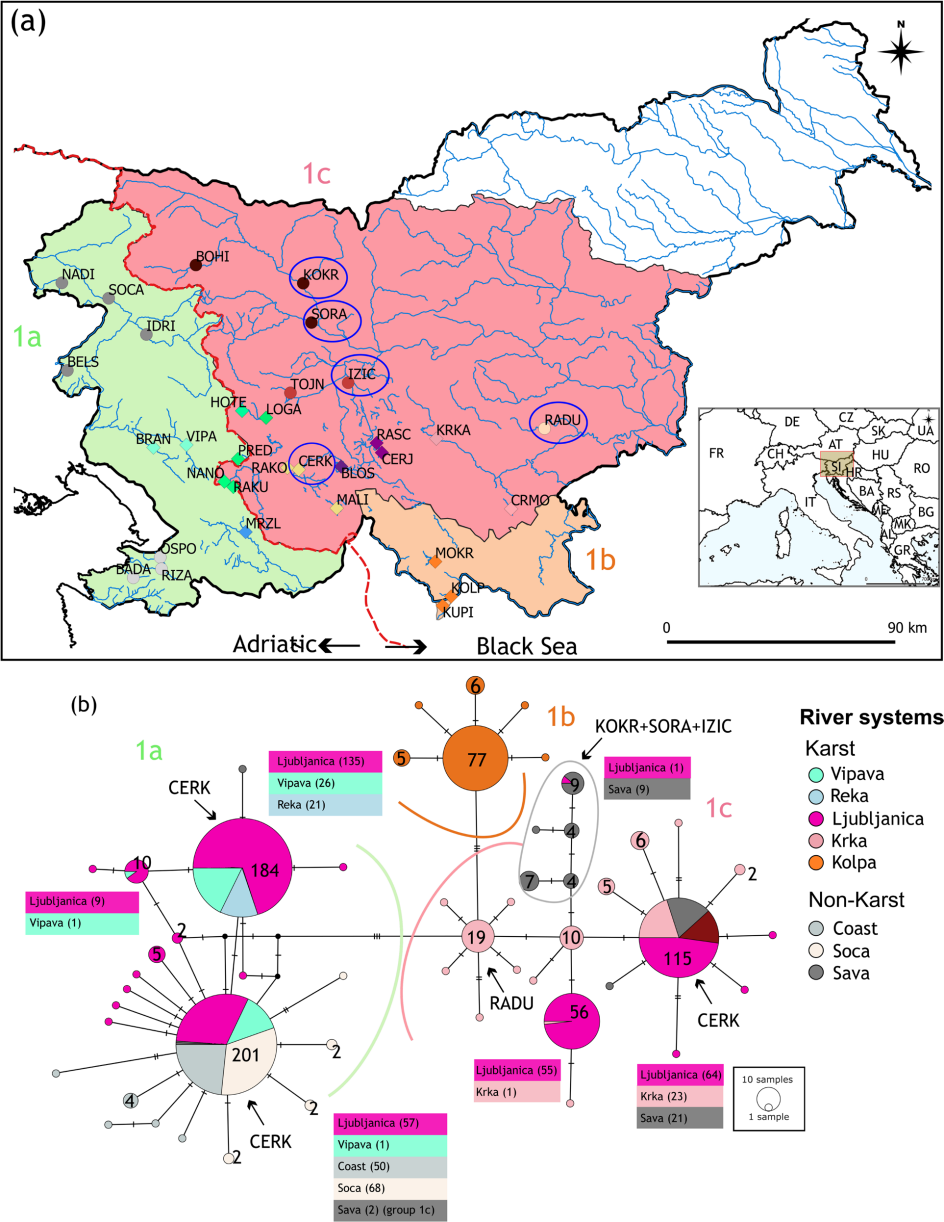
(a) Distribution patterns of the three main mitochondrial groups according to the findings of Reier, Kruckenhauser, et al. (2022): Main group 1a (pale green) spans the Adriatic drainage basin, crossing the border into the Black Sea basin; groups 1b (pale orange) and 1c (pale red) are situated in the Black Sea basin. Circled localities indicate sampling sites with unexpected grouping. (b) COI median‐joining haplotype network supporting the three main groups. Colors correspond to river systems. The numbers next to the haplotypes indicate the number of individuals found within each haplotype. Lines represent mutational steps, and black dots denote haplotypes that are missing from this dataset.

By adding the (mostly) non‐karst sampling sites collected in this study to the COI sequences from previous studies (Palandačić et al. 2015; Reier, Kruckenhauser, et al. 2022), three mtDNA main groups (1a–c) were confirmed in the NW Dinaric Karst and neighboring areas (Figure 2a, see also Introduction) using the COI MJ haplotype network. Groups 1a and 1c span karst and non‐karst sampling sites, while 1b is restricted to the karst area (Figure 2a,b). Notably, Ljubljanica RS sampling site (CERK) exhibits haplotypes from both 1a and 1c groups (encircled in Figure 2b, details in Figure S1). The sampling site from Krka RS (RADU) formed its own haplogroup(s) (highlighted in Figure 2b), while there are some sampling sites in non‐karst Sava RS (SORA, KOKR, and additional one specimen from sampling site IZIC (non‐karst part of the Ljubljanica RS); encircled in Figure 2b), which exhibit haplotypes a few mutational steps away from the main haplotype of the 1c group.

#### 3.2.2 Principal Component Analysis

**FIGURE 3:**
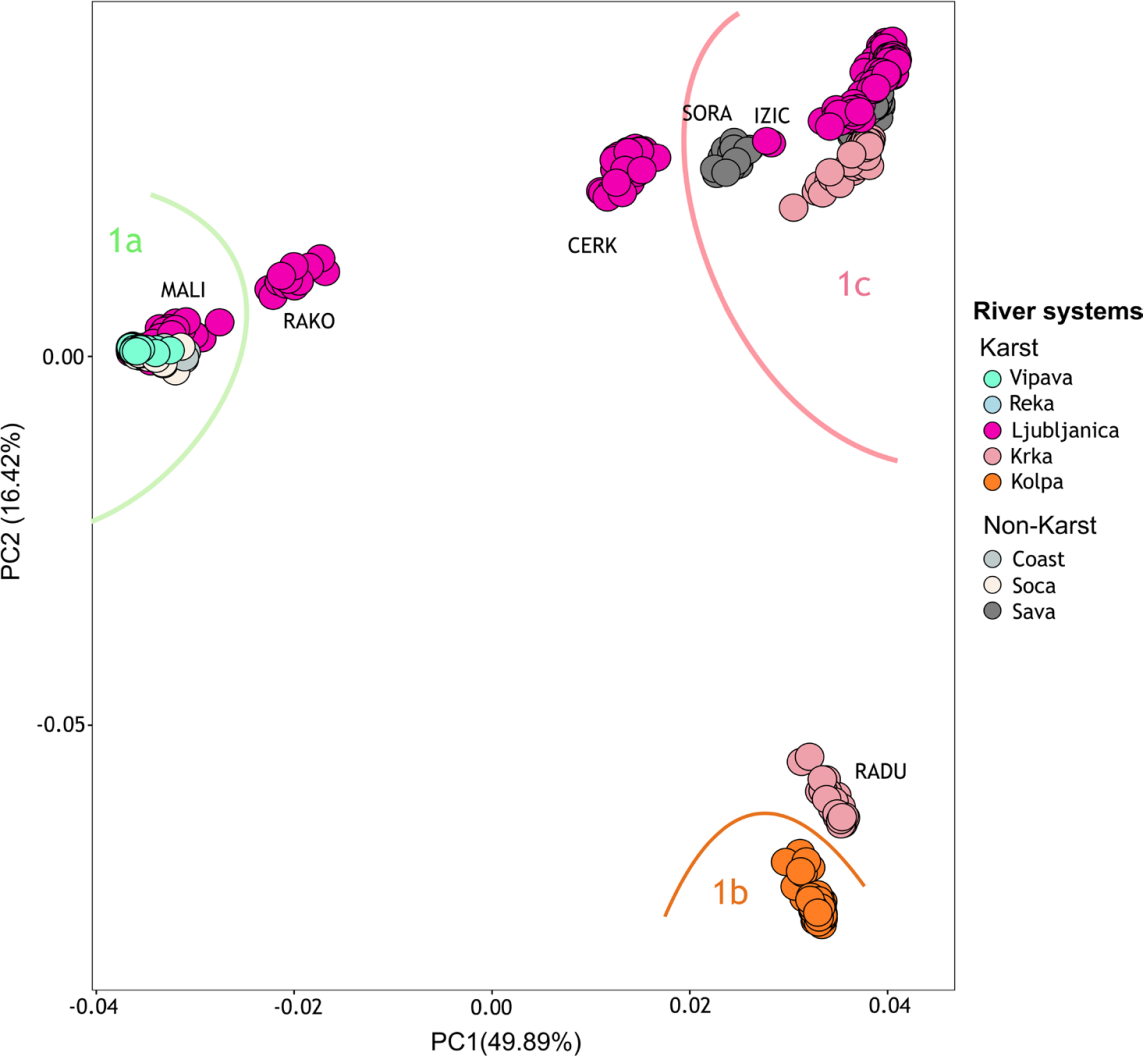
PCA, showing PC1 and PC2 with each dot representing an individual, with colors representing the river systems (RS). The three main groups (1a, b) are clearly separated. Admixed populations (RAKO, CERK; Ljubljanica RS) fall between groups 1a and 1c. Additional sub‐structuring is observed within the Ljubljanica RS (MALI, IZIC), Krka RS (RADU), and Sava RS (SORA), all of which stand out of their expected groupings.

The PCA based on SNPs from the ddRAD dataset confirmed the three main groups (1a–c; Figure 3), as well as additional structures seen in the mtDNA haplotype network (Figure 2b). Admixed populations from the mostly karstic Ljubljanica RS—specifically CERK and RAKO (both karst)—are positioned between groups 1a and 1c. Additional divergence was observed in the mostly karstic Krka RS, where the non‐karst sampling site RADU is separated in the PCA plot, and in the non‐karst Sava RS (sampling site SORA). These patterns are all highlighted in Figure 3.

#### 3.2.3 Effective Population Size History

Stairway plot analysis revealed a reduction in *N*_*e*_ across all three groups, with distinct temporal patterns. Group 1a experienced a decline 4000 years ago, followed by rapid expansion about 1000 years ago, with stability maintained for the last 800 years (Figure S2). Group 1b showed a sharp decrease in *N*_*e*_ approximately 35,000 years ago, followed by a stable period, and then, 15,000 years ago, a subsequent rapid decline to the lowest level 8000–6000 years ago, and a rapid recovery but to a comparatively lower level than prior to 35,000 years ago (Figure S2). In contrast, group 1c exhibited a more gradual, although still marked, decline in *N*_*e*_ 20,000 years ago, reaching a low 6000–5000 years ago, followed by a rapid recovery, although an ongoing decline began within the last 100 years (Figure S2).

### 3.3 Small‐Scale Population Structure

#### 3.3.1 Genetic Connectivity in Karst and Non‐Karst Systems

##### 3.3.1.1 ADMIXTURE Analysis

**FIGURE 4:**
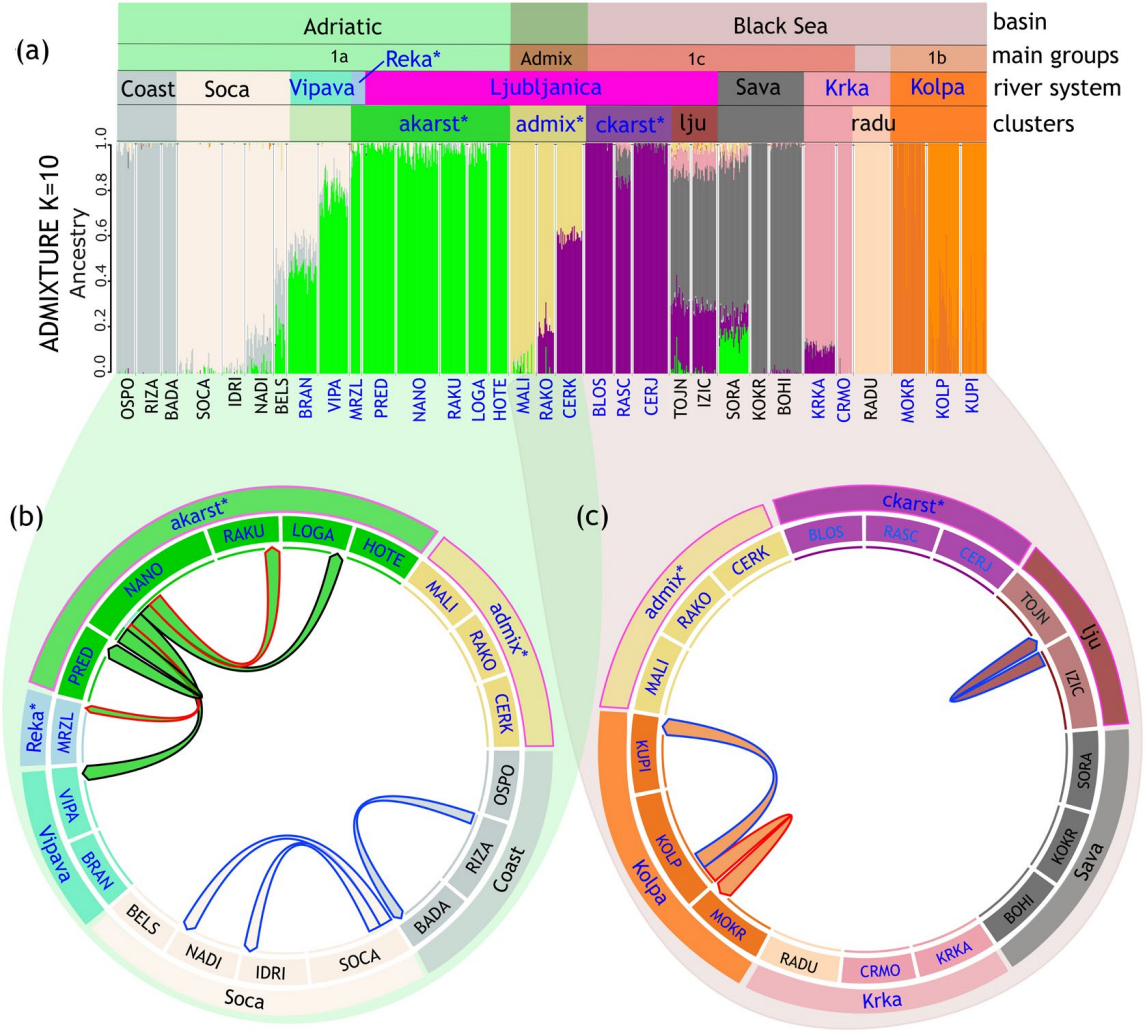
(a) ADMIXTURE bar plot at K = 10 showing the estimated admixture proportions for each sampling site. Sampling site names are listed below the bars, with blue indicating karst sites and black representing non‐karst sites. The colored bars represent estimated proportions of ancestry, corresponding to the number of ancestries (K). Labels above the bar plot indicate basin (Adriatic or Black Sea), main groups (1a–c or admix), river systems, and clusters within river systems (e.g., within Ljubljanica RS and Krka RS). (b, c) Bidirectional contemporary migration rates across river systems/clusters among (b) Adriatic sampling sites, and (c) among Black Sea sampling sites, as inferred from BA‐SNPs. Admix sampling sites (yellow) are included in both analyses. The Circos plots display only significant migration rates between sampling sites. Direction of arrow shows inward or outward gene flow between populations and are color‐coded based on connectivity type: Black outlines indicate underground connections revealed by tracing tests, blue outlines denote surface connections, and red outlines indicate no known connections (neither underground nor surface). RS/clusters with exclusively underground connections are marked with an asterisk.

To assess genetic structure and connectivity in karst and non‐karst RSs, ADMIXTURE analysis was performed. While no distinct lowest CV value was observed, a plateau emerged from *K* = 9 onward, indicating the presence of at least nine ancestral groups with small‐scale population structuring (Figure S3a). Since genetic groups identified by ancestry estimation programs do not always reflect biologically meaningful population structures (François and Durand 2010), K‐values should be interpreted carefully to avoid over‐interpretation (Caye et al. 2016). Although smaller‐scale structuring was detected by evalADMIX analysis (see Figure S3b), *K* = 10 was chosen for interpretation, as this level provided a biologically relevant balance between connectivity and differentiation across karst and non‐karst sampling sites, aligning with Hypothesis 1 (Figure 4a). Additional small‐scale structures at *K* = 13 are visualized in Figure S3c.

Following the hierarchical structure observed in previous analyses (mtDNA, PCA), ADMIXTURE analysis showed distinct genetic structuring of the three main groups (1a–c), with consistent differences in the proportions across regions (Figure 4a). Within each main group, admixture proportions reflected structuring by RSs, particularly in non‐karst sampling sites, where shared ancestry aligns with surface hydrology (e.g., Soča RS; Figure 4a).

Within the Ljubljanica RS, ADMIXTURE identified four distinct clusters, corresponding to different levels of karst connectivity:

- *akarst* (colored in green): Karst sampling sites (NANO, RAKU, PRED, HOTE, LOGA), connected only through underground water connections within the Ljubljanica RS (Figures 1b and 4a). Additionally, the MRZL sampling site from karst Reka RS also clustered within akarst, suggesting a genetic link between Reka RS and Ljubljanica RS, despite no known hydrological connection.
- *ckarst* (purple): Karst sampling sites (BLOS, CERJ, RASC) are also only connected through underground connections within Ljubljanica RS (Figures 1b and 4a). However, evalADMIX indicated more structure in this cluster not detected by ADMIXTURE at K = 10 (Figure S3b).
- *admix* (yellow): Karst sampling sites (RAKO, CERK, and MALI), connected underground within Ljubljanica RS and indicating admixture between Adriatic and Black Sea lineages (Figures 1b and 4a). Especially, RAKO and CERK exhibited mixed ancestry, suggesting underground hydrological connectivity with ckarst populations.
- *lju* (*dark red*): Non‐karst sampling sites (TOJN and IZIC), connected through surface connections within the Ljubljanica river (Figures 1b and 4a). Despite being classified as a non‐karst cluster, the Ljubljanica river originates from a karst spring with underground hydrological connections to other karst sampling sites within the Ljubljanica RS (Figure 1b), likely contributing to the admixture of lju between multiple ancestral groups.

Within the karst Vipava RS (VIPA, BRAN), an admixture between the non‐karst Soča RS and akarst is observed (Figures 1b and 4a). While Vipava RS maintains a surface connection with the Soča RS, it is also hydrologically linked to akarst through the underground.

The Krka RS is subdivided into karst sampling sites (KRKA, CRMO; colored in light pink, connected underground; Figure 4) and the non‐karst site RADU (colored in peach), which shows no shared ancestry with any other population (Figure 4a).

Within the surface connected Sava RS (non‐karst), evalADMIX results indicated more structure between sampling sites than revealed by *K* = 10 (Figure S3b,c).

##### 3.3.1.2 Contemporary Migration Rates

Significant migration rates revealed with BA3‐SNPs analysis were observed within and between karst sampling sites and RSs with underground connections as well as between non‐karst, surface‐connected sampling sites (see Figure 4b,c and Table S3):

- Within the akarst cluster, which has no surface connections but only underground connections. Some sampling sites exhibiting significant migration rates have no known underground connection (e.g., NANO and RAKU, see Figure 4b).
- Between akarst (Ljubljanica RS) and Vipava RS, two karst RSs, are connected underground.
- Between akarst (Ljubljanica RS) and Reka RS, two karst RSs with underground connections without known hydrological connections between them.
- Within the karst Kolpa RS, where surface connection exists, yet migration rates between sampling sites KOLP and MOKR indicate potential unknown underground pathways.
- Within non‐karst RSs, such as surface‐connected rivers in Soča RS, but also the Coast.
- Between the surface‐connected sampling sites in cluster lju (non‐karst part of Ljubljanica RS)

##### 3.3.1.3 *Pairwise* *F*_ST_

**FIGURE 5:**
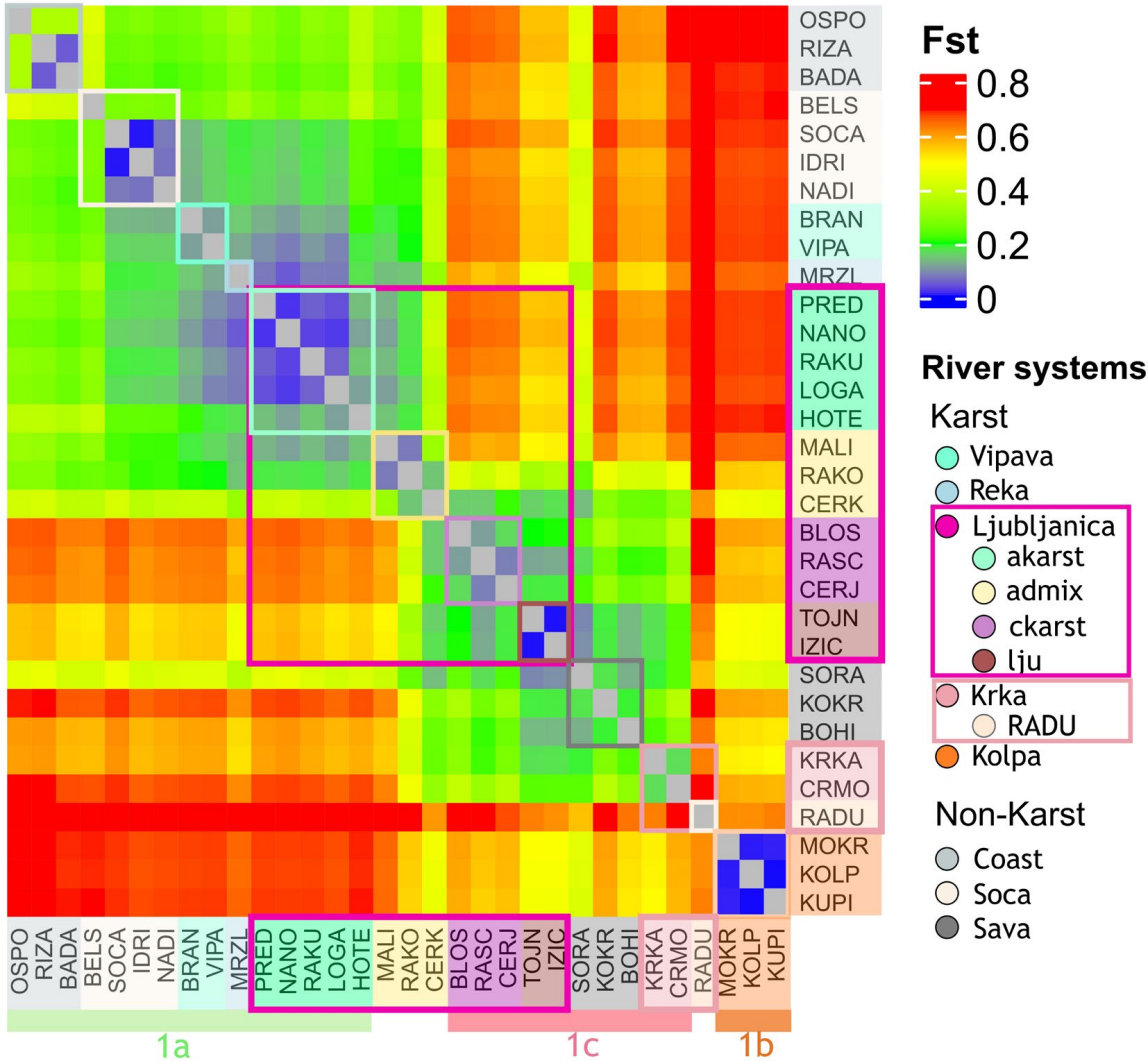
Pairwise *F*_ST_ values matrix using genome‐wide unlinked SNP data for *Phoxinus lumaireul*. Colors correspond to FST values, ranging from blue (≥ 0), green (≥ 0.2), yellow (≥ 0.4), and orange (≥ 0.6) to red (≥ 0.7). Each river system (RS) or cluster (within Ljubljanica and Krka RSs) is indicated by a colored square.

Low *F*_ST_ values (*F*_ST_ ≤ 0.04) were observed between all sampling sites within the non‐karst Soča RS and lju cluster (non‐karstic part of the Ljubljanica RS), which are linked through surface connections (Figure 5). Similarly, sampling sites belonging to the akarst cluster (Adriatic origin, Ljubljanica, and Reka RSs), despite being connected exclusively through underground connections, also exhibited low *F*_ST_ values (≤ 0.04), suggesting high genetic connectivity (Figure 5). In contrast, *F*_*ST*_ values were considerably higher (up to 0.2) among the ckarst cluster (Black Sea origin; Ljubljanica RS), likely due to more isolated underground connections. The non‐karst RADU sampling site (Krka RS) consistently showed the highest *F*_ST_ values, indicating pronounced differentiation from other populations (Figure 5).

##### 3.3.1.4 Genetic Diversity

The diversity metrics for each sampling site are given in Table S4. The genetic variation for Black Sea group 1c was overall highest, especially in the Ljubljanica RS clusters admix and lju. The non‐karst sampling site RADU from the Krka RS exhibited the lowest values. Values in main group 1b from the Black Sea were lower than those of the main group 1c. Diversity metrics in the main group 1a were generally low in all RSs/clusters, with the Coast sampling sites showing the lowest values. Inbreeding coefficients (*F*_IS_) were generally low with the exception of sampling site RASC (group ckarst), indicating some level of inbreeding or genetic structure.

Nucleotide diversity (*π*) differed significantly among main groups, with Adriatic sites showing the lowest values and sampling sites belonging to the main group 1c the highest. Pairwise Wilcoxon tests revealed significant differences between the main group 1a and all other groups, but not between admix and the two Black Sea groups (1b and c) or between the Black Sea groups 1b and 1c (Figure S4). π values did not vary significantly across RSs (all pairwise Wilcoxon *p* > 0.05, Figure S4). Populations with known underground connections revealed by tracing tests had significantly higher π values than surface‐connected populations (Figure S4). However, the karst status had no significant influence on π (Figure S4).

##### 3.3.1.5 Partitioning Genetic Variation

AMOVA was conducted to examine genetic variation among (i) the three main groups (see Section 3); (ii) RSs and clusters in Ljubljanica RS (akarst, admix, ckarst, lju) and sampling site RADU in Krka RS (see Section 3); and (iii) rivers with surface connections versus rivers with underground connections (see Table 1, Figure 1b). The largest proportion of variation was explained among the three main genetic groups (1a–c) (65.7%; Table 2), supporting the dominance of historical macroscale processes in shaping population structure. A significant proportion of variation was also explained among RSs and clusters (56%), indicating that contemporary river networks also contribute to genetic structuring. However, genetic variation between surface‐connected rivers (mostly non‐karst) and underground‐connected rivers (karst) was low and not statistically significant (*F*_CT_ = −0.042, *p* = 0.634; Table 2).

**TABLE 2:** Analysis of molecular variance (AMOVA) and degrees of freedom (df) for ddRADseq data among the three major groups identified in the present study (Figure 2a), the 12 clusters from the population genetic analyses, corresponding to the river systems, including the clusters of the Ljubljanica river system (Figure 1b), and the type of hydrological connectivity (underground or surface) corresponding to karst or non‐karst rivers (see Table 1 and Table S2 for details).

Source of variation	d.f.	Sum of squares	Variance component	Percentage variation	Fixation index	*p*
Among major groups (1a–c)	2	26,168.62	33.85	65.72	*F*_CT_ = 0.558	< 0.0001
Among sampling sites within major groups	21	2334.71	1.83	3.56	*F*_SC_ = 0.140	< 0.0001
Within sampling sites	1242	19,659.481	15.83	30.73	*F*_ST_ = 0.693	< 0.0001
Total	1265	48,162.81	51.52			
Among river systems/clusters	11	31,230.968	22.87	55.78	*F*_CT_ = 0.657	< 0.0001
Among sampling sites within river systems/clusters	16	2262.205	2.54	6.20	*F*_SC_ = 0.104	< 0.0001
Within sampling sites	1416	22,076.891	15.59	38.02	*F*_ST_ = 0.62	< 0.0001
Total	1443	55,570.064	41.0			
Between karst and non‐karst rivers	1	335.11	−1.41	−4.24	*F*_CT_ = −0.042	0.634 + −0.005
Among sampling sites within karst and non‐karst rivers	19	19,020.1	19.31	58.23	*F*_SC_ = 0.558	< 0.0001
Within sampling sites	1061	16,187.48	15.26	46.01	*F*_ST_ = 0.539	< 0.0001
Total	1081	35,542.690	33.158			

#### 3.3.2 Historical Influences on Population Structure

##### 3.3.2.1 Co‐Ancestry Analysis With fineRADstructure

**FIGURE 6:**
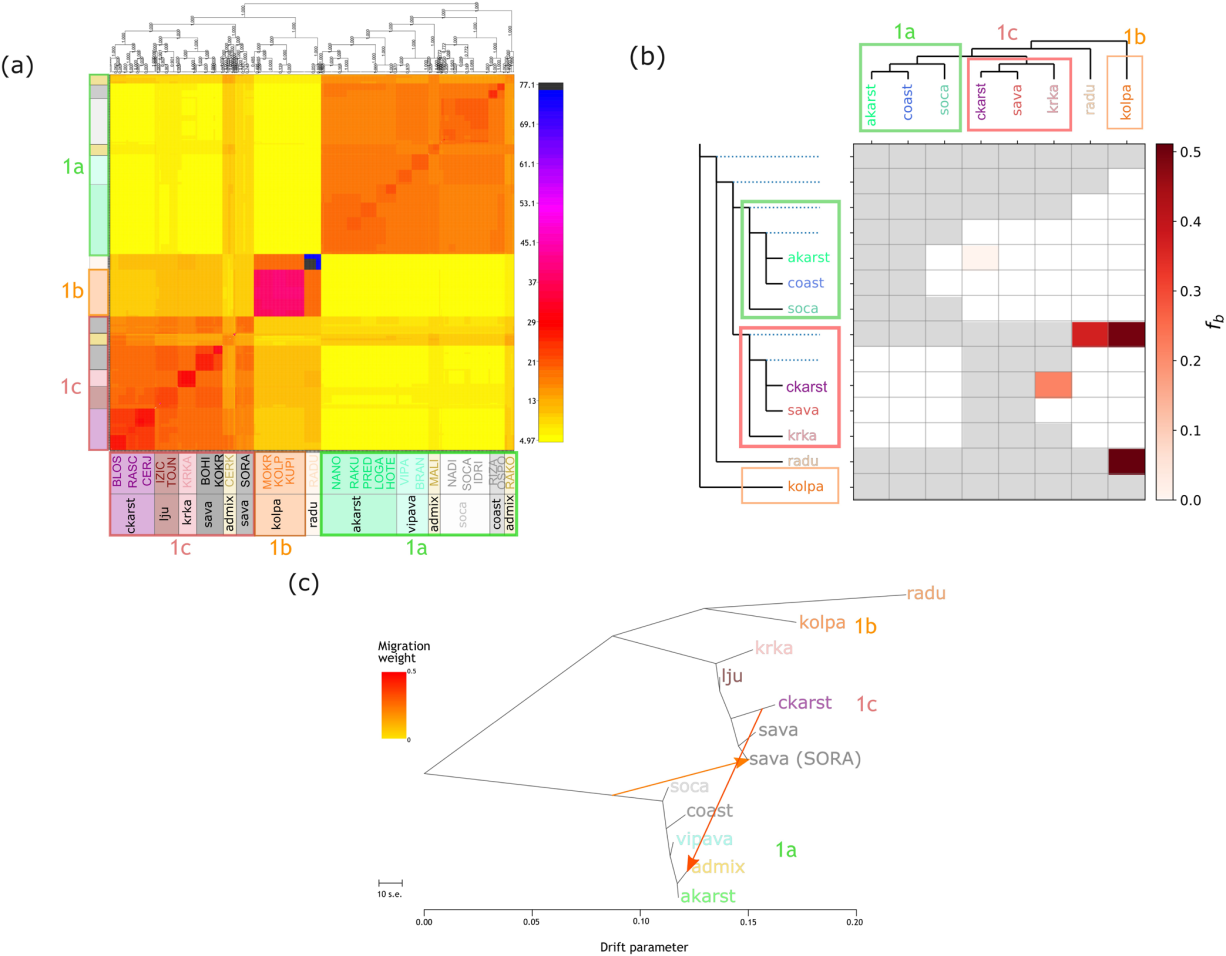
(a) Clustered fineRADstructure co‐ancestry matrix. Colors correspond to the level of co‐ancestry: Low, yellow; high, purple–blue–black. Colors of sampling site names correspond to river systems (RSs) and clusters within the Ljubljanica and Krka RSs. The main groups (1a–c) are indicated by rectangles around the corresponding RSs/clusters. (b) Heatmap of Dsuite *f*‐branch statistics highlighting potential introgression events (red squares). The phylogenetic tree, marked by main groups 1a (green), 1b (orange), and 1c (red), is shown along the x‐ and y‐axes. Gray squares denote trio combinations that cannot be tested due to topological constraints. (c) TreeMix maximum‐likelihood population tree based on unlinked genome‐wide SNPs illustrating two possible gene flow events depicted by arrows and colored by migration weight. The drift parameter is on the x‐axis, with the scale indicating 10× average SE of entries in the sample covariance matrix. The main groups (1a–c) are given next to the clades.

The fineRADstructure co‐ancestry analysis highlighted co‐ancestries within the main groups (1a–c), while also revealing smaller‐scale substructure among RSs (Figure 6a). Admixed co‐ancestry patterns were evident for specific sampling sites, indicating past genetic connectivity:

- CERK (Ljubljanica RS; admix cluster, karst), showing significant co‐ancestry with ckarst and, to a lesser extent, akarst.
- MALI and RAKO (Ljubljanica RS; also admix cluster, karst) showed predominantly ancestry of akarst, although co‐ancestry with ckarst was also indicated.
- The non‐karst sampling site SORA (Sava RS, non‐karst), while classified under main group 1c, also showed co‐ancestry with 1a, suggesting a historical connection.

Kolpa RS (karst, main group 1b) and RADU (non‐karst, Krka RS) exhibited the highest co‐ancestry patterns within their respective groupings when compared to other sites (Figure 6a).

##### 3.3.2.2 Detecting Historical Introgression With Dsuite

The *f*‐branch method to detect all possible population trios using *P. phoxinus* as an outgroup identified four introgression events between a given RS/cluster (Figure 6b, x‐axis) and branch *b* (*y*‐axis). The strongest signal of introgression (*f*_b_ = 0.51) was observed for karst Kolpa RS (main group 1b) and RADU (non‐karst part of Krka RS). Gene flow signals were also detected between lineages of main group 1c (encompassing ckarst (Ljubljanica RS), non‐karst Sava RS, and karstic Krka RS) and both Kolpa RS (*f*_b_ = 0.5) and RADU (*f*_b_ = 0.37; Krka RS), indicating historical connectivity. Within group 1c, the signals suggested gene flow between the karst sampling sites within ckarst (Ljubljanica RS) and Krka RS (excluding non‐karst RADU) (*f*_b_ = 0.22).

##### 3.3.2.3 Inferring Historical Relationships Using TreeMix

The TreeMix analysis revealed that two historical admixture events (*m* = 2) were optimal to explain more than 99.8% of the variance in the genetic data (Figure S5a). These two gene flow events indicated (Figure 6c):

- A genetic exchange from ckarst to admix (both Ljubljanica RS, karst) suggested past gene flow through underground connections within Ljubljanica RS.
- A gene flow event from an ancestor of main group 1a to the non‐karst SORA (Sava RS), despite no known hydrological connection.

However, the TreeMix residuals showed that the model fit for m = 2 may not fully capture certain aspects of the observed genetic data (see Figure S5b), indicating the possibility of additional admixture events, particularly between RADU (non‐karst part of Krka RS) and ckarst (Ljubljanica RS), Sava RS, and Krka RS, all belonging to main group 1c. Analysis of genetic drift among populations showed high values for the sampling site RADU (Krka RS) (Figure S5c).

## 4 Discussion

This study provides an in‐depth analysis of the genetic structure and diversity of the surface‐dwelling minnow *P. lumaireul*, with a focus on the influence of karst hydrology on shaping population connectivity. The findings, revealed through multiple complementary approaches—including mtDNA (Figure 2), PCA (Figure 3), and AMOVA (Table 2)—consistently confirmed that the genetic variation of *P. lumaireul* is primarily structured into three main genetic groups (1a–c), suggesting that Pleistocene glaciations and associated microrefugia had a greater impact on population structure than karstification. However, karst hydrology plays a key role at smaller spatial scales, facilitating genetic connectivity within and between RSs. The results largely support Hypothesis 1, showing that karst RSs generally facilitate genetic connectivity both within and between RSs, leading to higher genetic similarity among karst‐connected sampling sites (e.g., ADMIXTURE [Figure 4a]; BA3‐SNPs [Figure 4b], *F*_ST_ [Figure 5]). Yet, this pattern is not uniform across all karst systems, as some exhibit greater genetic isolation despite underground connections (BA3‐SNPs [Figure 4b], *F*_ST_ [Figure 5], evalADMIX [Figure S3b]). Besides contemporary hydrological networks, historical and intermittent connections have also influenced genetic structure. This supports Hypothesis 2, as genetic evidence reveals past admixture events and episodic connectivity, even where no present‐day hydrological links exist (Figure 1b and Figure 6a–c). Hypothesis 3 was only partially supported. While some subterranean pathways facilitated gene flow, others did not, despite hydrological connections confirmed by classical tracing tests (Figure 1b and Figure 4a,&,b). This suggests that not all underground water links act as functional dispersal corridors, possibly due to hydrological barriers, intermittent flow, or ecological constraints. These findings underscore the complexity of karst systems, where hydrological connections do not always mirror genetic connectivity.

### 4.1 Large‐Scale Population Structure Reflects Historical Rather Than Contemporary Hydrological Influences

Consistent with previous research (Palandačić et al. 2015, 2017, 2020; Reier, Kruckenhauser, et al. 2022), three main genetic groups (one with Adriatic basin origin [1a] and two with Black Sea basin origin [1b and 1c]) were identified based on mtDNA haplotypes and confirmed by SNP‐based analyses. Groups 1a and 1c spanned both karst and non‐karst RSs, while group 1b was restricted to the karst Kolpa RS, although previous research based on broader sampling areas identified mtDNA haplogroups belonging to 1b also in non‐karst areas (Palandačić et al. 2015; Vučić et al. 2018). The fact that the three main genetic groups encompass both karst and non‐karst sampling sites suggests that population structuring in *P. lumaireul* is not primarily shaped by ongoing karstification in the Dinaric Karst. If karstification processes were the main driver, we would expect genetic groupings to align more strictly with karst versus non‐karst hydrological systems. Instead, the observed genetic structure appears to reflect other historical processes, particularly Pleistocene glaciations and the presence of microrefugia, as seen in other aquatic taxa (Verovnik and Konec 2019). For example, Bravničar et al. (2021) connected the genetic structure of *Cottus* sp. with multiple microrefugia within the Upper Sava River with its pre‐Pleistocene origin (Bavec and Verbič 2011). Similarly, Pleistocene refugia may explain the presence of endemic invertebrate lineages in the Kolpa RS (e.g., Ivković and Plant 2015; Klobučar et al. 2013; Trontelj et al. 2005), in accordance with a separate group identified in this RS also in the present study (main group 1b). In contrast, the star‐shaped mtDNA haplotype network of group 1a suggests a single Adriatic refugium, followed by rapid recolonization. This scenario is further supported by significantly lower nucleotide diversity (*π*) in group 1a compared to the Black Sea groups (1b and 1c; Figure S4), likely indicating a recent bottleneck event rather than differences in hydrological connectivity. Such patterns are consistent with the genetic structure of other freshwater fish species in karst regions shaped by Pleistocene glaciations (e.g., Day et al. 2014; Perea and Doadrio 2015). Demographic analysis further supports this interpretation: Stairway plots show that all three groups originated during the Pleistocene, with group 1a emerging as the youngest and groups 1b and 1c with longer evolutionary histories (Figure S2), also aligning with previous estimates (Reier, Bogutskaya, et al. 2022; Reier, Kruckenhauser, et al. 2022). Historical declines in effective population size (*N*_e_) align with climatic shifts during the Würm glaciation and subsequent climatic fluctuations, including cold‐wet phases around 7500–7200 years and 5800–5200 years ago (Andrič et al. 2008; Heiri et al. 2014).

### 4.2 Underground Water Connections Facilitate Genetic Connectivity Within and Across Karst River Systems

Although the results largely support Hypothesis 1, indicating that karst underground connections facilitate genetic connectivity not only within but also between RSs, this pattern is not uniform across all karst systems.

Within those karst RSs where underground connectivity is well‐developed, genetic similarity was high (Figure 4a), *F*_ST_ values were low (Figure 5), and significant migration rates indicated ongoing gene flow between subterranean‐connected populations (Figure 4b). This was particularly evident in the akarst cluster (Ljubljanica RS), where underground gene flow maintains genetic continuity even across surface RS boundaries (e.g., Vipava and Reka RSs; Figure 4b). The pattern mirrors previous findings from Reier, Kruckenhauser, et al. (2022), where mitochondrial haplotype sharing between Reka, Vipava, and southwestern Ljubljanica RSs (corresponding to akarst) supported the role of karst underground water pathways in facilitating gene flow. The genetic continuity observed here further reinforces the idea that karst aquifers act as corridors for aquatic species, allowing gene flow despite surface hydrological boundaries (Konec et al. 2016).

Hereby, a particularly notable example is the unidirectional underground gene flow observed from sampling site NANO (akarst cluster, Ljubljanica RS; Figures 1b and 4a,b) to other sites. This sampling site is located within the sinking stream Pivka (Figure 1a,b), a hydrological junction where water drains underground and resurfaces in both the Adriatic and Black Sea drainage basins (Habič 1989; Konec et al. 2016). The observed significant migration rate from sampling site NANO to VIPA (Vipava RS; Figure 1, Figure 4b) suggests that this underground pathway has enabled gene flow across drainage basin divides. This pattern has not been observed in other surface‐dwelling freshwater fish species of the area (e.g., Bogutskaya and Zupančič 2010; Bravničar et al. 2021; Ketmaier et al. 2004). Thus, as suggested previously (Palandačić et al. 2015; Reier, Kruckenhauser, et al. 2022), *P. lumaireul* may utilize underground connections for dispersal while other surface‐dwelling fish species may not (Bravničar et al. 2021). A comparable crossing of the Adriatic–Black Sea divide in the NW Dinaric Karst region has been observed for subterranean fauna (e.g., Delić et al. 2022; Konec et al. 2016; Recknagel et al. 2023; Zakšek et al. 2009), further supporting the subterranean dispersal potential of *P. lumaireul*. An additional indication for the underground dispersal ability of *P. lumaireul* is its occurrence in caves (Reier, Bogutskaya et al. 2022; personal observation PT), reinforcing its potential as a genetic tracer for karst hydrology (see also below—Section 4.4).

However, this study showed that karst systems do not universally allow for connectivity. The ckarst cluster (Ljubljanica RS, main group 1c; Figure 1b), which is characterized by fewer known underground connections, exhibited greater genetic differentiation (Figure S3b,c), higher *F*_ST_ values (Figure 5) and absence of significant migration rates (Figure 4c) compared to the Adriatic akarst cluster (Figure 4b). This pattern highlights the role of hydrological isolation, which can occur in karst systems, consistent with studies on other taxa, such as cave bivalves (*Congeria*; Bilandžija et al. 2013), stone crayfish ( *Austropotamobius torrentium* ; Klobučar et al. 2013), and caddisflies (*Drusus* spp.; Previšić et al. 2014).

In contrast, non‐karst RSs exhibited genetic structuring aligned with surface hydrology. Low genetic differentiation within Soča RS and lju cluster (non‐karst part of Ljubljanica RS) supports the prediction that gene flow is mostly restricted to within‐river dispersal (Figure 4b,c), consistent with previous findings in other freshwater taxa, where genetic exchange occurs within RSs, but is limited between them (Hughes et al. 2012; Perea and Doadrio 2015). However, in some cases (e.g., non‐karst Sava RS), additional factors such as dams and anthropogenic modifications may limit connectivity in surface connected RSs (Figure 5, Figure S3b), as also revealed for the huchen ( *Hucho hucho* ) in the Upper Sava region (Snoj et al. 2022).

These results demonstrate that karst hydrology can enhance genetic connectivity both within and across RSs, but its effects are spatially variable. Connectivity depends on local hydrological features, seasonal fluctuations, and species‐specific dispersal capabilities, highlighting the complex interplay between subterranean and surface‐linked dispersal mechanisms.

### 4.3 Traces of Past Connectivity: Genetic Signatures of Historical Hydrological Events

The hypothesis that historical and occasional hydrological connections have influenced the population structure of *P. lumaireul* was supported by evidence of past gene flow detected through genomic analyses. These findings revealed historical water links that are no longer present in the current hydrological landscape. For example, historical introgression between Black Sea populations (e.g., group 1b [Kolpa] and an ancestor of group 1c; ckarst cluster [Ljubljanica RS] and Krka RS) was revealed by Dsuite analysis (Figure 6b). These genetic links correspond with historical connections previously proposed by other studies, which were based on the distribution patterns of subterranean fauna in this region (e.g., Sket 2002). Further, TreeMix analysis (Figure 6c) identified a major admixture event between the two karst groups (Adriatic akarst and Black Sea ckarst) within the Ljubljanica RS (today admix cluster, Figures 1b, 4a, and 6c), indicating past gene flow. This finding is further supported by co‐ancestry patterns (Figure 6a) and ADMIXTURE analysis results (Figure 4a), which provide additional evidence for historical admixture between these clusters and may have its origin in the redirection of the river Cerkniščica (CERK) in the late Pleistocene (Šušteršič et al. 2002).

Despite the absence of current hydrological connections, historical gene flow and shared ancestry between sampling sites belonging to main group 1a (Adriatic origin) and the SORA sampling site (Sava RS, main group 1c, Black Sea origin) are implied by fineRADstructure (Figure 6a) and TreeMix (Figure 6c). Thus, a historical water connection might have existed, further supported by some shared species between these two systems (Trontelj et al. 2005).

Overall, these findings demonstrate that episodic and historical hydrological changes have played a role in shaping the genetic structure of *P. lumaireul*, with genetic signatures persisting despite the loss of direct hydrological connections. Thus, as highlighted by Reier, Kruckenhauser, et al. (2022), the observed population structure reflects both contemporary hydrological networks (karst or non‐karst) as well as past hydrological processes.

### 4.4 Genomics as a Complement to Tracing Tests

Hypothesis 3 was partially confirmed as genetic tracing offers insights into the quality and functionality of hydrological connections that classical methods, such as dye or salt tracers, cannot fully capture. While tracing tests capture present‐day connections, genetic analyses can also offer a window into the past, occasional and functional hydrological connections, and reveal whether the connections are biologically relevant and passable for species such as *P. lumaireul*.

For example, the significant migration rates within the akarst cluster (e.g., between NANO and RAKU; Figures 1b and 4b) suggest connectivity not revealed by tracing tests. Such patterns likely arise from episodic events, such as fluctuations in water levels and extreme floods, which may have facilitated dispersal under specific circumstances (e.g., Zakšek et al. 2009), as observed previously in lowland rivers (e.g., Hughes 2007). Since no direct underground water links are currently known, the connection between the Ljubljanica and Reka RSs appears to be occasional or indirect (Reier, Kruckenhauser, et al. 2022), although geological evidence suggests that these systems were repeatedly connected during the Pleistocene (Habič 1989).

In contrast, some hydrological connections detected through tracing tests show no genetic connection (e.g., some sampling sites from akarst and admix clusters to lju cluster within Ljubljanica RS; cf. Figure 1b), potentially due to the quality of connections (e.g., Grabovšek and Turk 2010), that are passable for water and dye, respectively, but not for fish. These findings align with prior research highlighting the limitations of classical tracers, which often provide only “snapshots” of connectivity under specific hydrological conditions (e.g., Goldscheider 2015; Tobin et al. 2024). Furthermore, the variable permeability and flow‐path organization of karst systems, driven by dissolution processes and episodic hydrological events, complicate interpretations of connectivity and emphasize the need for complementary approaches (Goldscheider et al. 2020; Morrissey et al. 2020). Genetic tracing addresses these challenges by revealing biologically relevant connectivity, and occasional and past hydrological connections that are often missed by traditional hydrological methods.

However, genomic data also revealed that hydrological connections alone cannot fully explain the observed patterns of genetic diversity and differentiation. The AMOVA results (Table 2) showed that large‐scale structures (65.7%) and RSs (56%) are the dominant factors in genetic differentiation. While karst hydrology plays a role in shaping connectivity at a small scale (Figure S4), it does not outweigh the past climatic and geological events that structured populations of *P. lumaireul* into the three main genetic groups and within RSs. These findings are consistent with previous studies demonstrating that historical developments of RSs play a key role in shaping local population structures (e.g., Chiba et al. 2015; Wu et al. 2016). Despite these limitations, *P. lumaireul* emerges as a promising genetic tracer for karst aquifers due to its robust population structure and ability to reflect hydrological dynamics. Combining genomic approaches with classical methods enhances our understanding of karst hydrology and RS histories, supporting the conservation of these complex ecosystems.

### 4.5 Preserving Karst Biodiversity: Opportunities for Future Research

Approximately 14% of the world's land surface is covered by karst, spanning diverse climatic zones and geological conditions (Williams 2008), which prevent broad generalizations from specific case studies. However, all karst systems share common vulnerabilities such as habitat fragmentation, pollution, and the impacts of climate change. Understanding the population patterns, drivers of genetic diversity, and species‐specific dispersal limitations in these regions is therefore essential for developing effective conservation strategies. Future research should aim to refine methodologies for comparing genetic and hydrological connectivity, incorporate high‐resolution environmental data, and expand the taxonomic scope to include multiple species. Such approaches will enhance our understanding of the interplay between ecological, hydrological, and geological factors in shaping biodiversity.

This study represents—to the authors' knowledge—the first comprehensive investigation of the population structure and dispersal capabilities of a surface‐dwelling fish species within a karst hydrological network. It emphasizes the importance of conserving freshwater habitats within the Dinaric Karst, not only to protect *P. lumaireul* populations but also to preserve the remarkable biodiversity these dynamic ecosystems support.

## Conflicts of Interest

The authors declare no conflicts of interest.

## Supporting information


Data S1.

**Table S1.** Summary of all samples analyzed in this study, including main group (1a–c), river systems, sampling sites, geographic coordinates, SRA archive accession numbers, NCBI COI accession numbers, and data sources.

## Data Availability

All relevant data are deposited in public databases. Raw sequence reads of ddRAD data are deposited in the sequence read archive (SRA; BioProject PRJNA1251853), while COI sequence data are deposited in the NCBI Nucleotide Database, and accession numbers are specified in Table S1 together with accession numbers of downloaded COI sequences. The bioinformatic code for ddRAD data processing is available on Github (https://github.com/susiArdeidae/Phoxinus‐in‐Dinaric‐Karst/), and the corresponding input files and codes for population genetic analyses are stored in the NHM data repository (https://datarepository.nhm.at/; https://doi.org/10.57756/58udvj).
