# Supplementary material for: Factors Beyond Karstification Have Shaped the Population Structure of a Surface‐Dwelling Minnow (Phoxinus lumaireul) Able to Disperse Underground
Source: Evol Appl. 2025 May 12;18(5):e70104. doi: 10.1111/eva.70104 (PMC12067393; doi:10.1111/eva.70104)
Supplement: Supplementary file 1 — Data S1. [file EVA-18-e70104-s001.pdf]

Factors beyond karstification have shaped the population structure of a surface-dwelling minnow (*Phoxinus lumaireul*) able to disperse underground

Susanne Reier[1,2], Peter Trontelj[3], Luise Kruckenhauser[1,4], Martin Kapun[4], Alexandra Wanka[4], Anja Palandačić[2,3]

Supplementary material

1. List of Tables

Table S1. Provided as separate Excel file.

Table S2. Sampling sites structured by major group, river system or cluster, and whether on karst or not, used for hierarchical AMOVA, with results reported in Table 2.

**Major group**		**River system or cluster**		**Underground or surface river**	
group 1a	HOTE	coast	OSPO	Underground (karst)	HOTE
	NANO		RIZA		LOGA
	PRED	soca	NADI		NANO
	RAKU		IDRI		PRED
	LOGA		SOCA		RAKU
	OSPO	vipava	BRAN		CERK
	RIZA		VIPA		RAKO
	NADI	akarst	HOTE		MALI
	IDRI		NANO		BLOS
	SOCA		PRED		CERJ
	BRAN		RAKU		RASC
	VIPA		LOGA	Surface (non-karst)	NADI
group 1b	MOKR	admix	CERK		KOKR
	KOLP		RAKO		IZIC
	KUPI		MALI		IDRI
group 1c	KOKR	ckarst	RASC		SOCA
	BOHI		CERJ		SORA
	IZIC		BLOS		TOJN
	SORA	lju	IZIC		BRAN
	TOJN		TOJN		BOHI
	KRKA	sava	KOKR		VIPA
	RASC		BOHI		
	CERJ		SORA		
	BLOS	krka	KRKA		
		radu	RADU		
		kolpa	MOKR		
			KOLP		
			KUPI		

Table S3. Contemporary migration rates among sampling sites based on BA3-SNPs analysis. Migration rates are shown with standard error (SE) and p-values, categorized by recipient and source populations, with details on surface or underground hydrological connections. Migration rates are considered significant if the p-value is below 0.05. Surface connections are indicated as "surface," hydrological pathways confirmed by tracing tests as "underground," and pathways with no known connection are marked as "No known connection." The table separates results for populations within the Black Sea and Adriatic basins.

	**Recipient**	**Source**	**Migration Rate (± SE)**	**p-Value**	**Surface/underground connection**
**Black Sea**					
karst	KUPI	KOLP	0.2102 (± 0.0247)	≈ 0.0	surface
karst	MOKR	KOLP	0.2286 (± 0.0212)	≈ 0.0	No known connection
Non-karst	TOJN	IZIC	0.1837 (± 0.0277)	3.32×10^{-11}	surface
**Adriatic**					
Non-karst	BADA	RIZA	0.1504 (± 0.0283)	5.50×10^{-8}	surface
Non-karst	IDRI	SOCA	0.1743 (± 0.0265)	2.60×10^{-11}	surface
Non-karst	NADI	SOCA	0.1800 (± 0.0624)	0.002	surface
karst	LOGA	NANO	0.1802 (± 0.0265)	5.35×10^{-12}	Underground (indirect)
karst	MRZL	NANO	0.0916 (± 0.0268)	3.18×10^{-4}	No known connection
karst	PRED	NANO	0.2172 (± 0.0214)	≈ 0.0	Underground
karst	RAKU	NANO	0.2006 (± 0.0243)	≈ 0.0	No known connection
karst	VIPA	NANO	0.1986 (± 0.0331)	1.97×10^{-9}	Underground

Table S4. Summary of genetic diversity metrics for each sampling site. Sites are grouped by main genetic group, river system/cluster, and include observed heterozygosity (H_O), expected heterozygosity (H_E), nucleotide diversity (Pi), and inbreeding coefficient (F_{IS}). Sampling sites within Ljubljanica river system are indicated with respective cluster name (akarst, admix, ckarst, lju), and RADU from the Krka river system is representing a distinct sampling site with unique genetic characteristics.

Sampling site	main group	River system/cluster	H_O	H_E	*Pi*	F_{IS}
BADA	1a	Coast	0,066	0,065	0,068	0,0052
OSPO	1a	Coast	0,062	0,062	0,064	0,0065
RIZA	1a	Coast	0,065	0,065	0,067	0,0044
BELS	1a	Soca	0,058	0,056	0,058	0,0013
IDRI	1a	Soca	0,097	0,096	0,098	0,0084
NADI	1a	Soca	0,091	0,092	0,094	0,0085
SOCA	1a	Soca	0,093	0,095	0,096	0,0101
BRAN	1a	Vipava	0,095	0,096	0,097	0,0081
VIPA	1a	Vipava	0,095	0,098	0,100	0,0143
MRZL	1a	Reka	0,093	0,084	0,089	-0,0113
NANO	1a	akarst	0,102	0,103	0,104	0,0070
RAKU	1a	akarst	0,096	0,096	0,098	0,0060
PRED	1a	akarst	0,097	0,099	0,100	0,0106
LOGA	1a	akarst	0,098	0,100	0,103	0,0128
HOTE	1a	akarst	0,085	0,083	0,086	0,0022
MALI	admix	admix	0,124	0,123	0,125	0,0087
RAKO	admix	admix	0,225	0,221	0,228	0,0096
CERK	admix	admix	0,293	0,296	0,302	0,0242
BLOS	1c	ckarst	0,215	0,216	0,219	0,0137
RASC	1c	ckarst	0,243	0,270	0,278	0,1498
CERJ	1c	ckarst	0,231	0,225	0,229	-0,0060
IZIC	1c	lju	0,275	0,281	0,287	0,0314
TOJN	1c	lju	0,282	0,288	0,295	0,0346
BOHI	1c	Sava	0,235	0,240	0,244	0,0241
KOKR	1c	Sava	0,200	0,201	0,207	0,0174
SORA	1c	Sava	0,298	0,303	0,308	0,0288
CRMO	1c	Krka	0,188	0,189	0,196	0,0208
KRKA	1c	Krka	0,234	0,241	0,245	0,0294
RADU		RADU	0,043	0,044	0,045	0,0053
KOLP	1b	Kolpa	0,118	0,129	0,131	0,0400
KUPI	1b	Kolpa	0,119	0,128	0,131	0,0356
MOKR	1b	Kolpa	0,117	0,127	0,129	0,0340

2. List of Figures

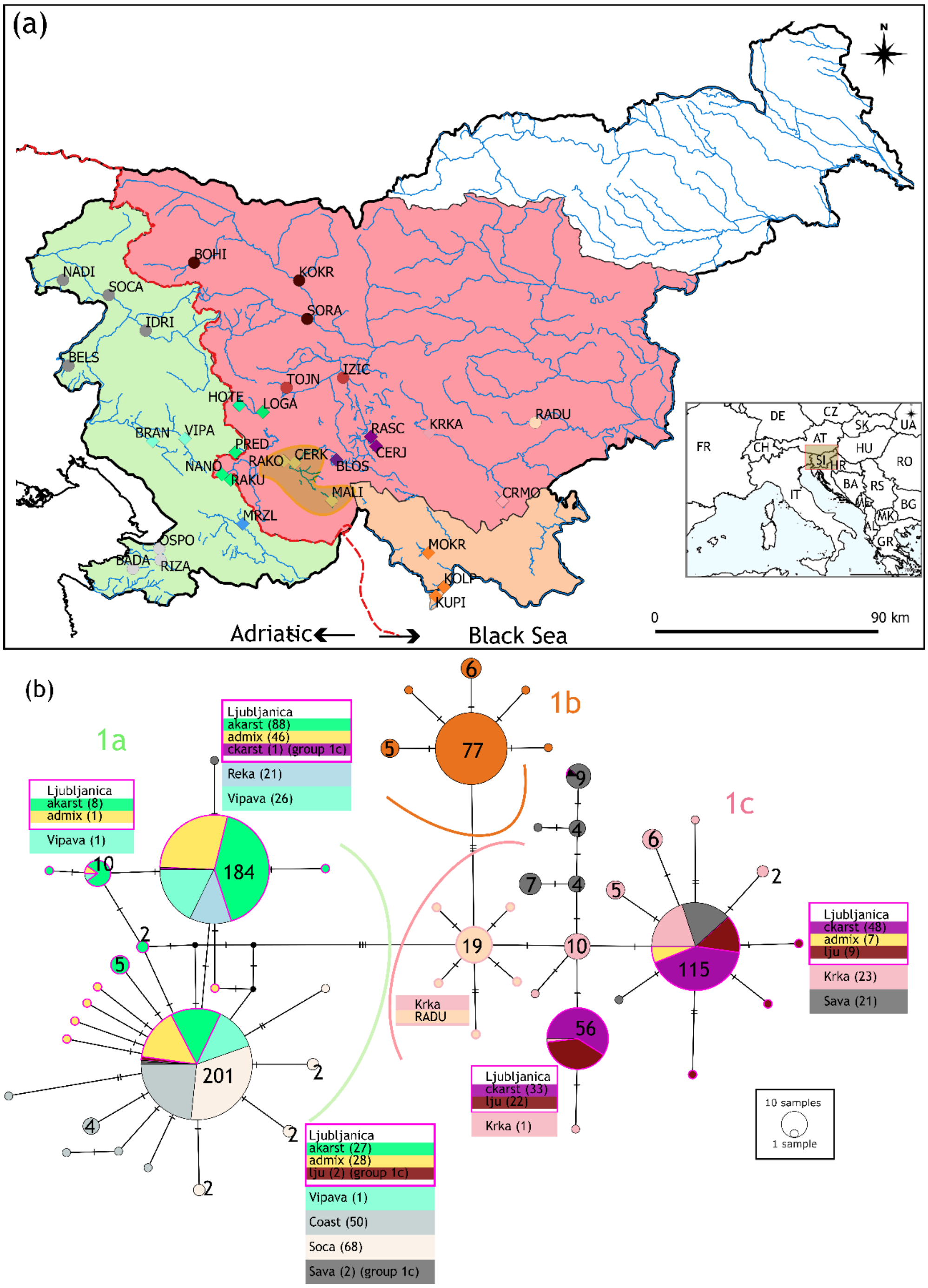

Fig. S1 (a) Distribution patterns of the three major mitochondrial groups according to the findings of Reier, Kruckenhauser et al. (2022): main group 1a (pale green) spans the Adriatic drainage basin, crossing the border into the Black Sea basin; groups 1b (pale orange) and 1c (pale red) are situated on the Black Sea basin. The admix cluster, as revealed by ADMIXTURE analysis is colored in yellow. (b) COI Median-Joining haplotype network supporting the grouping into three main groups. Colors correspond to river systems (RS) and cluster within Ljubljanica RS (akarst, admix, ckarst, lju) and Krka RS (RADU). The numbers next to the haplotypes indicate the number of individuals found within each haplotype. Lines represent mutational steps, and black dots denote haplotypes that are missing from this dataset.

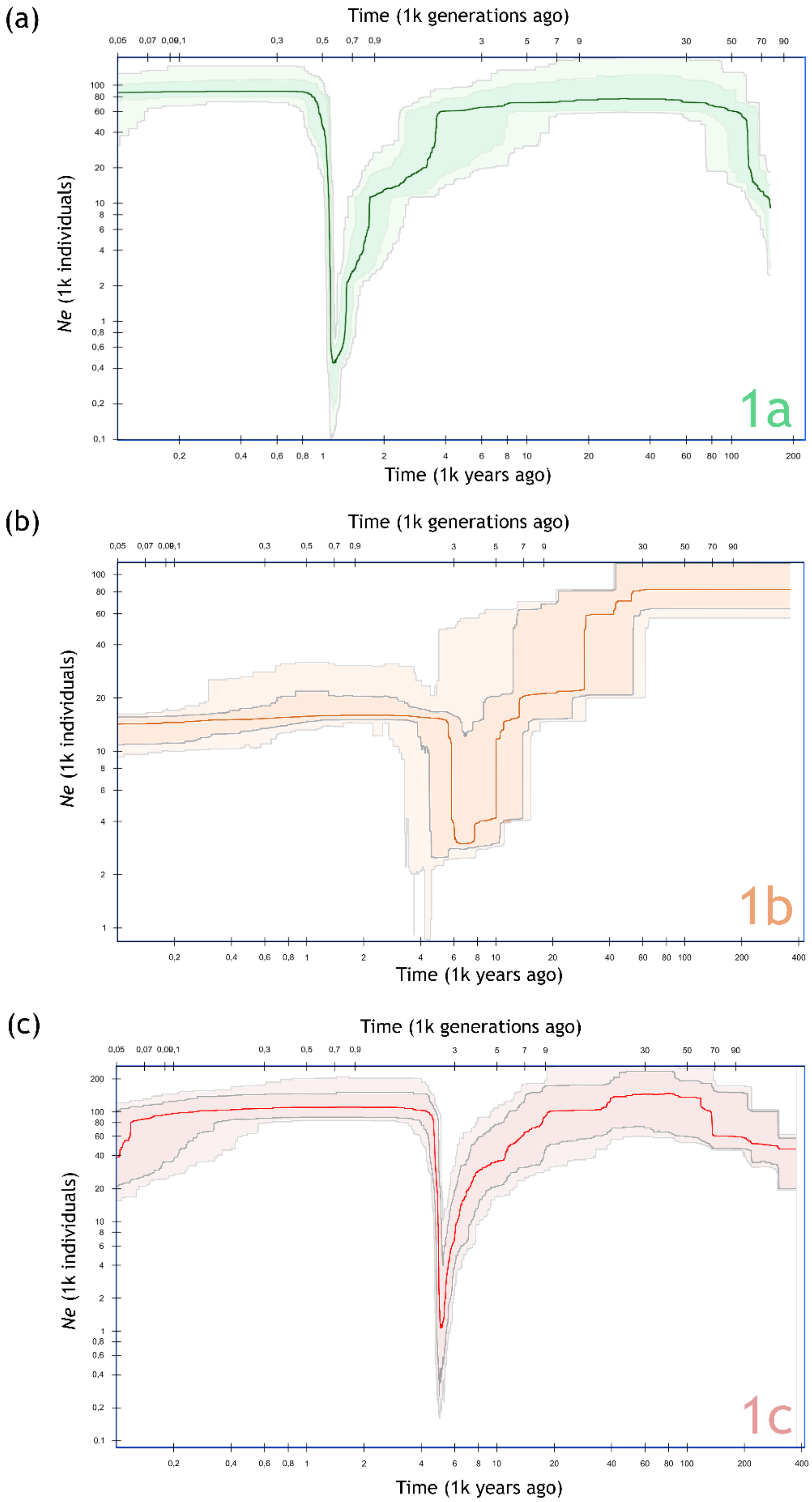

Fig. S2. Stairway plots showing the changes in effective population size (N_e) for each of the main groups (1a-c). **(a)** Group 1a. **(b)** Group 1b. **(c)** Group 1c. Y-axis indicates N_e per 1,000 individuals. Lower x-axis shows the time in thousands

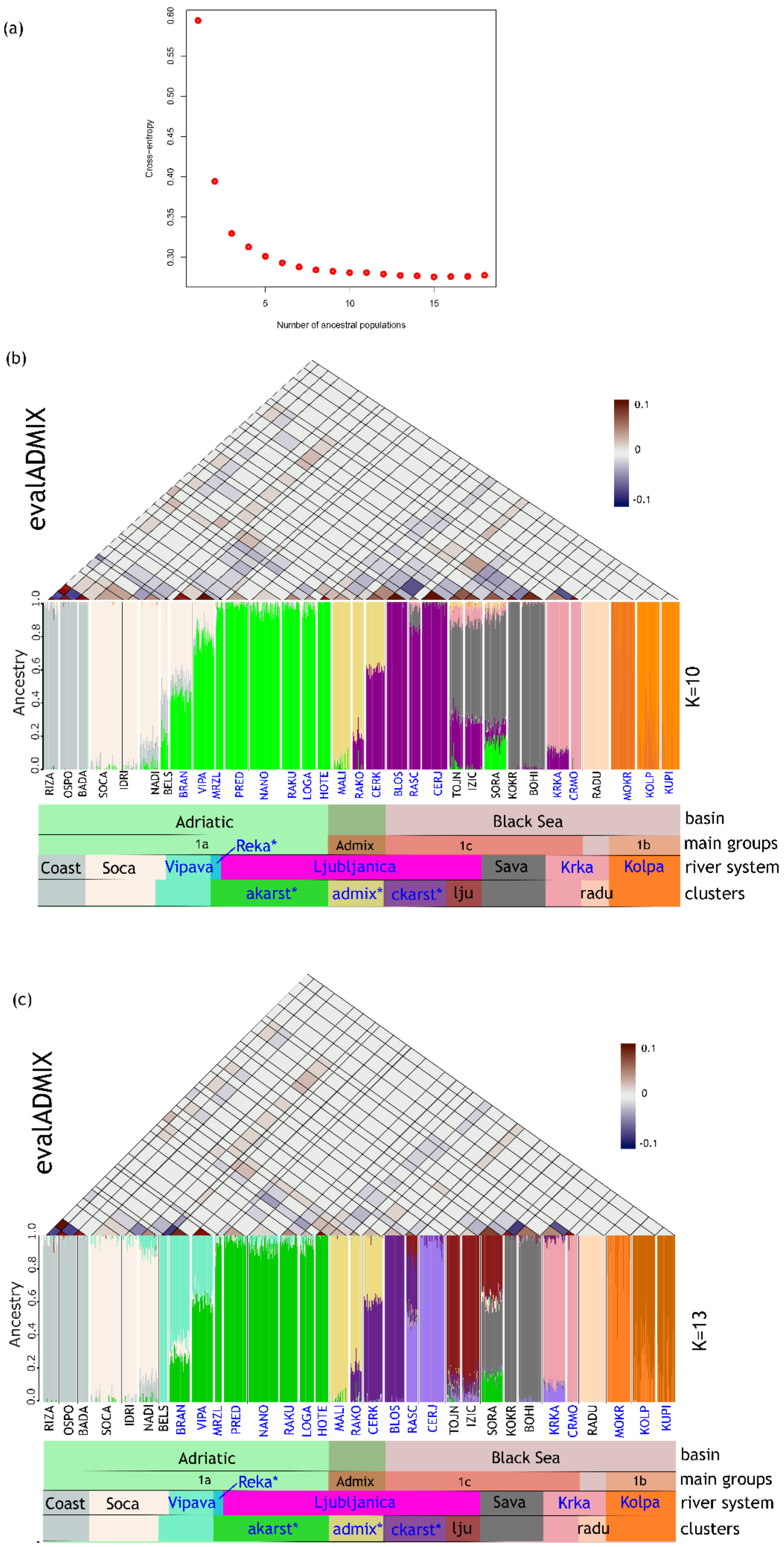
(a)
Cross-entropy
0.30
0.35
0.40
0.45
0.50
0.55
0.60
5
10
15
Number of ancestral populations
(b)
evalADMIX
0.1
0
-0.1
Ancestry
0.0
0.2
0.4
0.6
0.8
1.0
K=10
RIZA OSPO BADA SOCA IDRI NADI BELS BRAN VIPA MRZL PRED NANO RAKU LOGA HOTE MALI RAKO CERK BLOS RASC CERJ TOJN IZIC SORA KOKR BOHI KRKA CRMO RADU MOKR KOLP KUPI
Adriatic
Black Sea
basin
1a
Reka*
Admix
1c
1b
main groups
Coast
Soca
Vipava
Ljubljanica
Sava
Krka
Kolpa
river system
akarst*
admix*
ckarst*
lju
radu
clusters
(c)
evalADMIX
0.1
0
-0.1
Ancestry
0.0
0.2
0.4
0.6
0.8
1.0
K=13
RIZA OSPO BADA SOCA IDRI NADI BELS BRAN VIPA MRZL PRED NANO RAKU LOGA HOTE MALI RAKO CERK BLOS RASC CERJ TOJN IZIC SORA KOKR BOHI KRKA CRMO RADU MOKR KOLP KUPI
Adriatic
Black Sea
basin
1a
Reka*
Admix
1c
1b
main groups
Coast
Soca
Vipava
Ljubljanica
Sava
Krka
Kolpa
river system
akarst*
admix*
ckarst*
lju
radu
clusters

Fig. S3 **(a)** Plot of cross-validation (CV) error from K=1 to K=18 for ADMIXTURE analysis. **(b & c)** ADMIXTURE bar plot at **(b)** K=10 and **(c)** K=13 showing the estimated admixture proportions for each sampling site. Sampling site names are listed below the bars, with blue indicating karst sites and black representing non-karst sites. The colored bars represent estimated proportions of ancestry, corresponding to the number of ancestries (K). Labels above the bar plot indicate basin (Adriatic or Black Sea), main groups (1a–c or admix), river systems, and clusters within river systems (e.g., within Ljubljanica RS and Krka RS). Pairwise correlations of residuals, as estimated by evalAdmix, are shown above the respective ADMIXTURE barplots, ranging from −0.1 to 0.1 on the color scale.

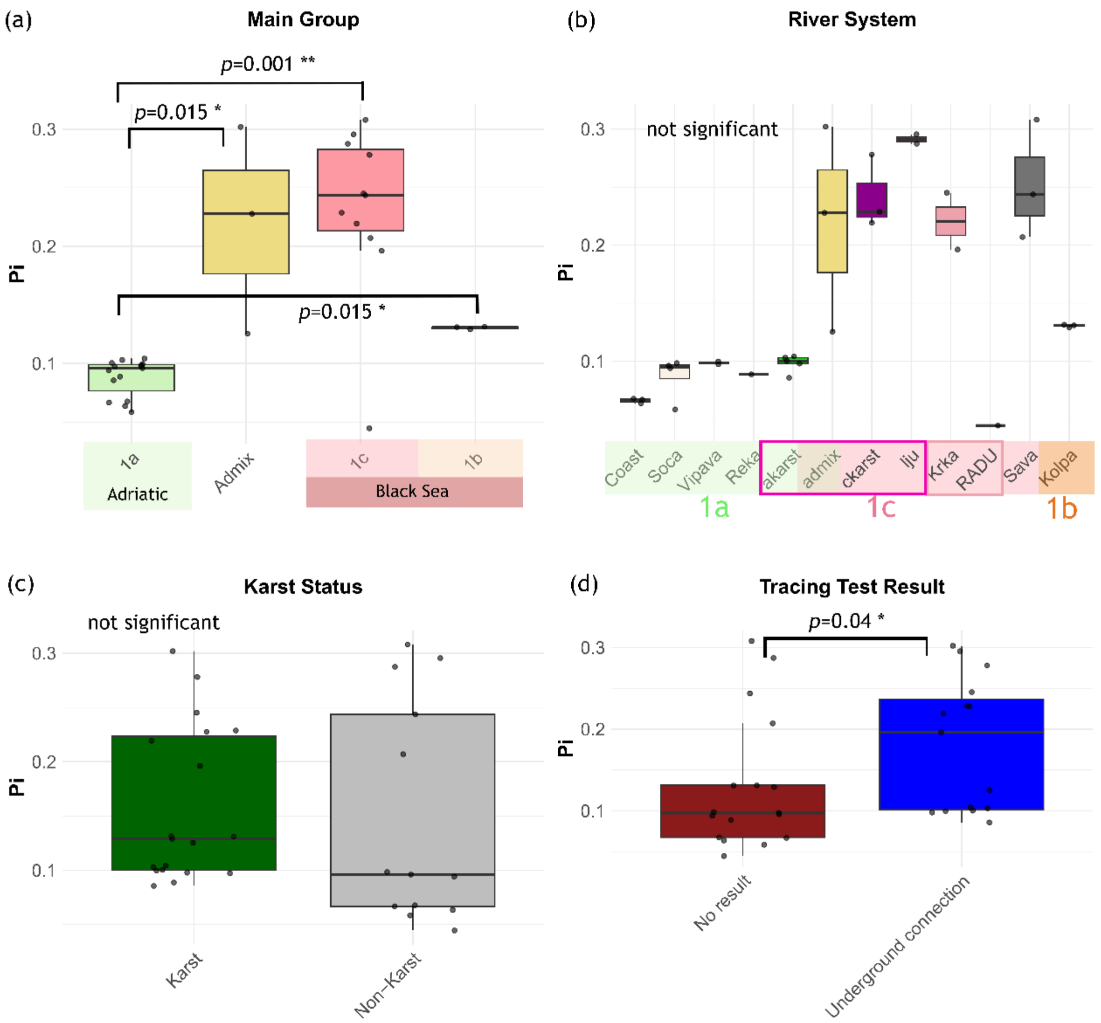

Fig. S4 Boxplots showing the comparison of nucleotide diversity (Pi) values across different grouping categories based on Wilcoxon rank sum tests: **(a)** Pi values grouped by the three main genetic groups (1a, 1b, 1c) and admix. Significant differences are indicated between groups, with *p*-values shown (*p*=0.015 and *p*=0.001). Adriatic (1a) populations display the lowest diversity, while Black Sea (1b, 1c) and admix groups show higher diversity. **(b)** Pi values across river systems and clusters within the Ljubljanica RS (akarst, admix, ckarst, lju) and Krka RS (RADU). No significant differences are observed between river systems/clusters. **(c)** Pi values grouped by karst status (karst vs. non-karst). No significant differences are detected between the two categories. **(d)** Pi values categorized by the presence or absence of known underground connections, as revealed by tracing tests. Sites with underground connections show significantly higher Pi values (*p*=0.04). Significant *p*-values are indicated by * (<0.05) and ** (<0.01).

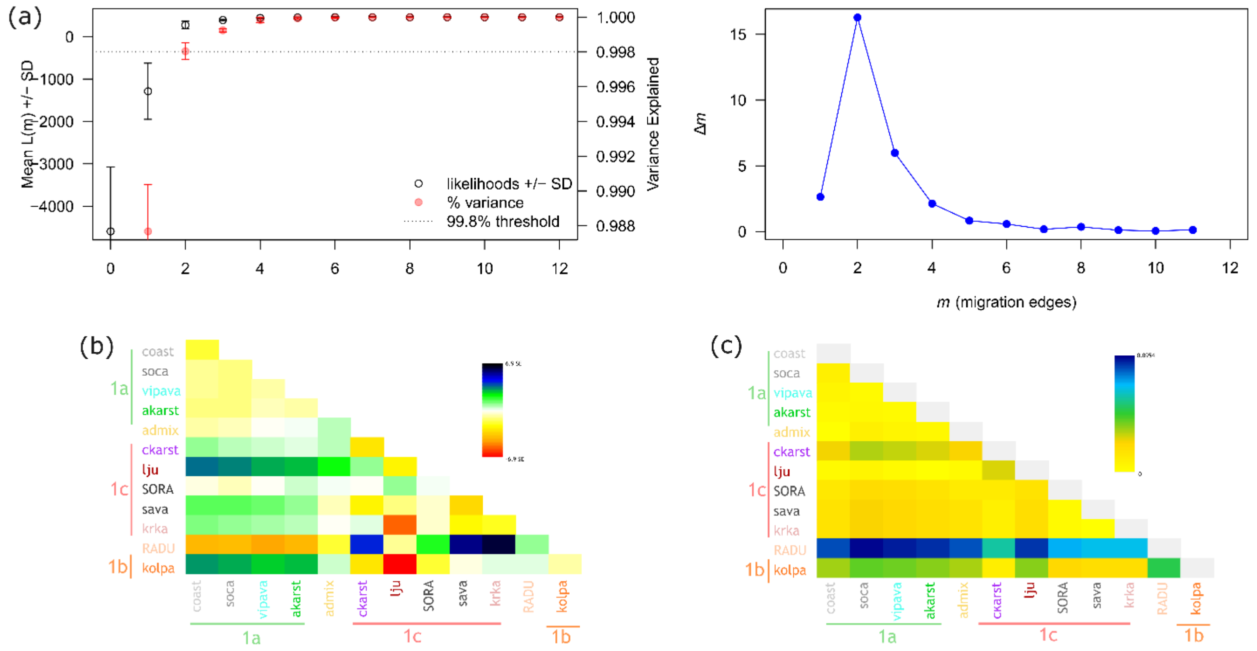

Fig. S5 (a) OptM output for TreeMix analysis. The plot on the left depicts the mean and standard deviation (SD) across 100 iterations for the composite likelihood L(m) (left axis, black circles) and proportion of variance explained (right axis, red circles). The 99.8% threshold (horizontal dotted line) is recommended by Pickrell & Pritchard (2012). The plot on the right shows the second-order rate of change (Δm) across values of m, with m=2 being the chosen migration edge for the TreeMix analysis. **(b)** Heatmap of residual fit from the tree, with positive residuals (blue–black) suggesting population pairs more closely related than in the best-fit tree and indicating potential admixture events. Negative residuals (red–orange) suggest populations less closely related than in the best-fit tree. **(c)** Heatmap representing amount of genetic drift between each population pair, with colors proportional to drift intensity, ranging from yellow (minimum) to dark blue (maximum).
